# Geography, Environmental Conditions and Life History Shape Patterns of Within‐Population Phenotypic Variation in North American Birds

**DOI:** 10.1111/ele.70244

**Published:** 2025-11-09

**Authors:** Viviane Zulian, Casey Youngflesh

**Affiliations:** ^1^ Department of Biological Sciences Clemson University Clemson South Carolina USA

**Keywords:** climate change, eco‐evolutionary processes, environmental variability, hierarchical Bayesian modelling, intraspecific trait variation, ITV, life history, phenotypic traits

## Abstract

Intraspecific variation is a fundamental component of biodiversity, shaping species interactions and coexistence dynamics. While numerous mechanisms have been proposed to shape the degree of phenotypic variation within species, many remain largely untested or poorly explored at broad spatial and taxonomic scales. Using data from nearly 200,000 bird captures from 99 species across North America, we investigated hypothesized drivers of within‐population phenotypic variation, using body mass and wing length as traits of interest. The magnitude of observed phenotypic variation was modulated by a combination of geographic, environmental, and life history factors. This was true whether considering differences in within‐population phenotypic variation within or among species. The impact of these non‐mutually exclusive mechanisms has resulted in substantial variation in the observed magnitude of within‐population phenotypic variation. These results provide empirical evidence for a set of long‐standing hypotheses regarding the processes that regulate observed patterns of this understudied, but important, component of biodiversity.

## Graphical abstract

Intraspecific variation is a fundamental component of biodiversity, shaping species interactions and coexistence dynamics. While numerous mechanisms have been proposed to shape the degree of phenotypic variation within species, many remain largely untested or poorly explored at broad spatial and taxonomic scales. Using data from nearly 200,000 bird captures from 99 species across North America, we show that phenotypic variation within populations was modulated by a combination of geographic, environmental, and life history factors, with support for a set of long‐standing hypotheses.

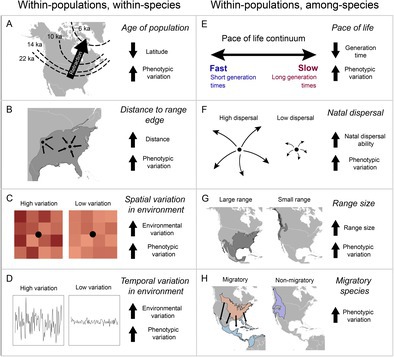

## 1 Introduction

Phenotypic variation within species plays an important role in shaping ecological (Des Roches et al. 2018) and evolutionary processes (Bolnick et al. 2011). While studies of biodiversity often focus on variation *among* species (Downing and Leibold 2002; Hillebrand and Matthiessen 2009; Pigot et al. 2020), variation *within* species can be as large as these interspecific differences (Albert et al. 2010). Intraspecific differences can mediate competition within ecological communities, both within and among species, which may have important consequences for fitness (Laughlin and Messier 2015) and coexistence dynamics (Fussmann et al. 2007; Palkovacs and Post 2009). Identifying the processes that drive these observed patterns of variation provides a mechanism by which to understand the eco‐evolutionary processes that shape ecological systems (Raffard et al. 2019) and ultimately, to predict how species are likely to respond to global change (Moran et al. 2016).

While many mechanisms have been hypothesized to regulate intraspecific phenotypic variation, these have been either untested or poorly explored at large spatial and taxonomic extents. We focus on the magnitude of phenotypic variation among individuals *within populations* of a given species (rather than among populations), and how this varies across space and among species. This is one component of what is referred to as Intraspecific Trait Variation, or ITV (Violle et al. 2012; Westerband et al. 2021).

**FIGURE 1:**
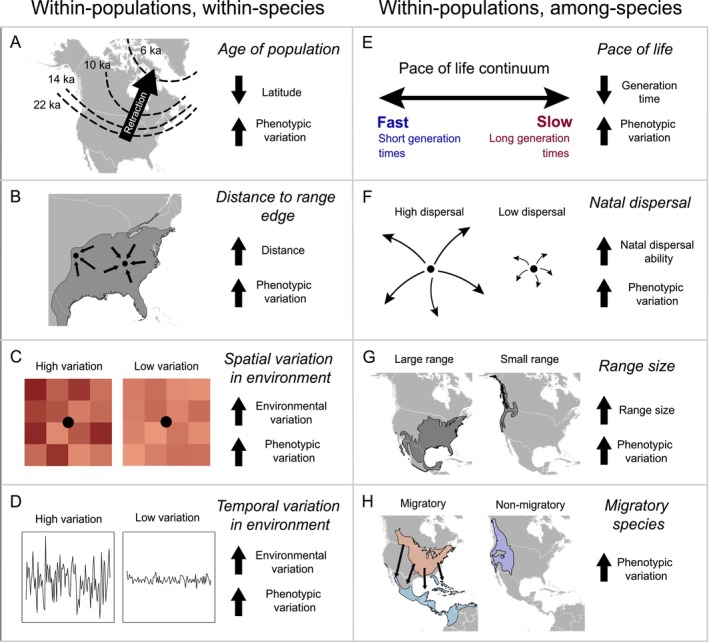
Multiple non‐mutually exclusive mechanisms have been hypothesized to impact the degree of within‐population phenotypic variation in North American birds. Within species, greater variation is expected in populations that are (A) located at lower latitudes (as higher latitude populations are likely to have been established more recently following glacial retreat after the Last Glacial Maximum) (Hewitt 2000), (B) are located at the center of a species range (Eckert et al. 2008; Pironon et al. 2017), (C) experience greater spatial (Vernham et al. 2023), and (D) temporal variation in environmental conditions (Bull 1987; Schultz 1989). Among species, greater phenotypic variation is expected in species that, (E) have a faster pace‐of‐life (characterised by shorter generation times) (Schultz 1989; Wright et al. 2019), (F) have greater natal dispersal ability (Bowler and Benton 2005), (G) have larger range sizes (Slatyer et al. 2013), and (H) that are seasonally migratory (Webster et al. 2002). For A, numbers represent the approximate extent of the ice sheet at different periods of time. For B and C, points represent hypothetical locations at which phenotypic data were collected. For H, the orange polygon represents the breeding range, the blue polygon represents the non‐breeding range, and the purple polygon represents the year‐round range.

The degree of phenotypic variation observed within a population is expected to vary both across a species range, as well as among species. Within a given species, greater phenotypic variation among individuals might be expected in populations that were established further into the past (Hewitt 2000) (Figure 1A). Newly established populations are founded by a subset of individuals of a given species, which may possess only a portion of the phenotypic variation found in the source population. Spatial gradients in phenotypic variation might therefore be expected with directional range expansions, which often result from a series of successive founding events (Slatkin and Excoffier 2012). Populations located further from range margins are expected to exhibit greater phenotypic variation as well, as these populations will experience a higher influx of phenotypic variation from other populations (Eckert et al. 2008; Pironon et al. 2017) (Figure 1B). Finally, the magnitude of environmental variation, over both space (Figure 1C) and time (Figure 1D), has been suggested to shape phenotypic variation within species. Greater spatial variation may indicate a greater set of ecological niches to be exploited by a larger set of phenotypic traits (Cordero and Epps 2012; Vernham et al. 2023). Greater temporal environmental variation may favour populations with greater phenotypic variation, as selection pressures that a species experiences might vary over time (Bull 1987; Schultz 1989; Yamamichi et al. 2023).

Considering differences among species, it has been proposed that greater phenotypic variation (i.e., average within‐population variation for a species) should be expected in species that have a faster pace of life (Schultz 1989; Wright et al. 2019) (Figure 1E). For ‘fast‐paced’ species, which live for relatively short periods of time, theoretical models show that selection should favour a range of phenotypes suited to many possible environments. ‘Slow‐paced’ species, on the other hand, can reproduce on more occasions and should thus produce a narrower range of phenotypes (Schultz 1989). Species with greater natal dispersal capacity are also expected to exhibit greater phenotypic variation, as this is expected to result in a greater influx of phenotypic variation into populations compared to species where natal dispersal is quite limited (Ronce 2007) (Figure 1F). Species with larger ranges, being exposed to a more diverse set of habitat conditions (Slatyer et al. 2013) (Figure 1G), as well as migratory species, being exposed to different conditions across both their breeding and non‐breeding grounds (Webster et al. 2002) (Figure 1H), might also be expected to exhibit greater phenotypic variation.

**FIGURE 2:**
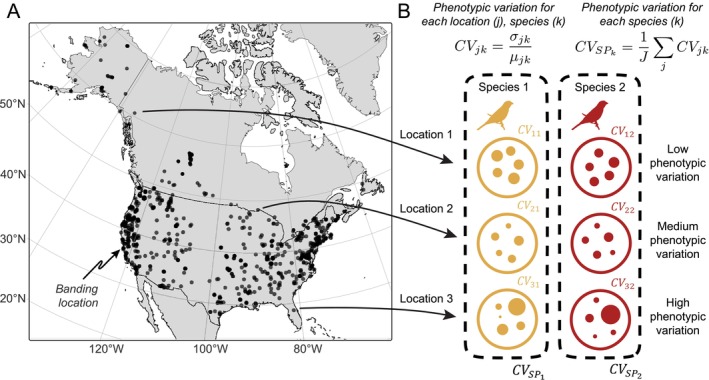
Estimates of within‐population phenotypic variation for 99 bird species across North America were derived data from the MAPS bird banding project. (A) Black points show the 875 locations (banding sites) from which phenotypic data was collected. (B) Measurements of individual birds were used to estimate the coefficient of variation, ${\mathrm{CV}}_{\mathrm{jk}}$ (Equation 4), where $\sigma_{\mathrm{jk}}$ is the standard deviation and $\mu_{\mathrm{jk}}$ is the mean of a given trait for species *k* at location *j*. Averaging CV across all locations for each species, we derived species‐specific estimates of within‐population (i.e., among individual) phenotypic variation, ${\mathrm{CV}}_{{\mathrm{SP}}_{k}}$ (Equation 7).

Leveraging a collection of data sources, including individual‐level morphological information from 99 bird species, species‐level traits and environmental variables, we assessed the degree to which these hypothesized mechanisms have shaped observed patterns of intraspecific phenotypic variation in North American birds. Our morphological data were derived from 197,794 individual bird captures from 875 locations across North America (Figure 2A) during the breeding season (April–August) over the last 30 years (DeSante et al. 2019). Our synthetic approach included a flexible, hierarchical Bayesian framework to account for multiple sources of uncertainty in the data, variation in responses among species, and phylogenetic non‐independence. We use both body mass and wing length (i.e., unflattened wing chord) as our phenotypic traits of interest, and quantify the degree of variation in each trait for each species in a given location using the coefficient of variation (CV, Figure 2B), to account for the fact that larger variation is expected when the mean of a given trait is larger (Pélabon et al. 2020). Our analysis of this spatially and taxonomically extensive data set (Figure 2) demonstrated that intraspecific phenotypic variation is driven by a combination of geographic, environmental, and life history processes, with support for multiple long‐standing, non‐mutually exclusive hypotheses regarding the mechanisms that regulate patterns of biodiversity.

## 2 Materials and Methods

### 2.1 Morphological Data

We used morphological data from the Monitoring Avian Productivity and Survivorship (MAPS) program, a large‐scale, long‐term bird banding initiative established by The Institute for Bird Populations (DeSante et al. 2004). Sampling locations were distributed across North America, each following a standardised protocol during the breeding season of North American birds (April to August) (DeSante et al. 2019). Data were obtained from 875 locations (Figure 2A), sampled between 1989 and 2018, though not all locations were active for the entire period. Each location consisted of 6–20 mist‐nets operated 6–12 times per year. For each captured or recaptured bird, the body mass (in grams) and the wing length (i.e., unflattened wing chord, in millimetres) were measured, and the sex and age recorded (DeSante et al. 2019; Pyle 1997). While other traits are, no doubt, equally relevant, we focus specifically on body mass and wing chord as these were readily available from this data source and measured according to a standardised protocol.

We restricted our data set to include only banding locations and species with at least 15 individuals captured, and only species where individuals were captured at five locations or more and across at least five degrees of latitude to ensure that data were available over a reasonably large area. To avoid confounding effects of age and sex on morphological measurements, we filtered the data to include only adult males, as females show substantial variation in body mass depending on the stage of reproduction (due to the contribution of eggs to total body mass) (Dumas et al. 2024; Meijer et al. 1994; Nwaogu et al. 2017). While body mass of males may also vary across the breeding season, this should not bias the results, given data collection was stratified across the breeding season, following the MAPS protocol (DeSante et al. 2019). If an individual was captured multiple times in a season, we used only the data collected in the first capture. Fewer than 7% of individuals were captured more than three times over the course of the study. Outliers, defined as any value falling outside of 5 median absolute deviations (MAD) (following Youngflesh et al. 2022), were also excluded, as these likely resulted from measurement or recording errors. In total, our data set comprised measurements from 197,794 individual captures across 99 species.

### 2.2 Mechanisms Shaping Within‐Population Variation Across Species' Ranges

We assessed several processes that might drive differences in phenotypic variation across species' ranges. First, we considered the latitude (*Lat*) of each banding station (range: 28.1–69.4 degrees N) as a proxy for time of colonisation following the Last Glacial Maximum (Hewitt 2000). As ice sheets retreated northward with increasing temperatures, suitable habitat also shifted, facilitating the establishment of more northerly populations.

Second, we considered the effect of distance to range edge (*DistEdge*). We measured the shortest distance (in kilometres) of each location to the species' breeding range edge. To account for the effect of small lakes and rivers in range maps, we removed all gaps smaller than 10‐km that were entirely within the species range. This was done by creating a 10‐km buffer around the range edge, calculating the distance of each location to that buffered edge, and then subtracting 10‐km from that distance. Range maps were downloaded from the BirdLife database (BirdLife International and Handbook of the Birds of the World 2022). Locations located outside the range map were assigned a distance of zero.

Third, we considered spatial variability in environmental conditions (*SpatVar*) at a location, sometimes referred to as geodiversity (Vernham et al. 2023). We used variation in cumulative annual productivity, measured by Normalised Difference Vegetation Index (NDVI) from the Dynamic Habitat Indices (DHI) (Radeloff et al. 2019) available at 1‐km spatial resolution as our environmental metric. NDVI results from different biotic and abiotic factors, such as temperature and precipitation, which directly influence bird community composition and richness (Fairbairn et al. 2025; Hurlbert and Haskell 2003). For each location, we extracted the average cumulative annual productivity across all years for each 1‐km cell within a 10‐km radius buffer. A 10‐km buffer was used around each capture location to capture the broader area used by individuals around each location. We then calculated the standard deviation of the cross‐year averages across all cells and divided this by the average of the cross‐year averages across all cells within each buffer to obtain a CV for each location. We used CV instead of the standard deviation, as productivity is ratio scale data (i.e., it has a meaningful 0).

Lastly, we considered temporal variation (*TempVar*) in environmental conditions at each location. As above, we used the temporal variation in productivity (NDVI) derived from the published DHIs (Radeloff et al. 2019) as our environmental variable of interest. Within a 10‐km radius buffer around each location, we calculated the temporal CV as the standard deviation of each cell across years divided by the mean across years. We used the mean CV of all cells within the buffer for each location. We log‐transformed *DistEdge*, *SpatVar*, and *TempVar* values, as all were right skewed.

### 2.3 Differences in Within‐Population Variation Among Species

We were also interested in processes that shape differences in phenotypic variation among species. First, we considered the effect of generation time (*GenTime*), a key indicator of the pace of life of a given species (Healy et al. 2019). These were derived from published values from Bird et al. (2020). Generation times for species ranged from 1.8 to 4.3 years.

Second, we considered the natal dispersal ability of each species, as indicated by the hand‐wing index (*HWI*) (Tobias et al. 2020), a measure of the elongation of the wing. This metric is a reliable predictor of natal dispersal distance across a large number of species (Arango et al. 2022; Chu and Claramunt 2023; Weeks et al. 2022). HWI values were derived from published values from Tobias et al. (2022) and ranged from 11.4 to 53, with higher values indicating higher natal dispersal capacity.

Third, range size (*RangeSize*) of each species (in km^2^) was calculated using published range maps (BirdLife International and Handbook of the Birds of the World 2022). For migratory species, we combined both the breeding and non‐breeding ranges to determine the total range size. Species' range sizes varied from ~180,000 to ~28,000,000 km^2^. We log‐transformed *GenLength*, *HWI*, and *RangeSize* values as all were right skewed.

Finally, we considered migratory status (*MigStatus*). Each species was categorized as either a migrant (1) or non‐migrant (0) and based on species range maps. Species were classified as migrants if their breeding range differed from their non‐breeding range. While this ignores the complexities of migratory behavior, such as cases where some individuals in an area or even entire populations of a species migrate while others do not, this approach broadly captures propensity to migrate for these species. Of the 99 species in our dataset, 88 were classified as migrants.

### 2.4 Estimating Location‐ and Species‐Specific Variation

For each location and species, we quantified the degree of within‐population variation in both body mass and wing length using the CV as our standardised measure of variation (Pélabon et al. 2020). For each trait, we modelled the log of the observed trait (trait) for each individual i, location j, and species k, as:

(1) $${\text{trait}}_{\mathrm{ijk}}\sim N\left(\mu_{\mathrm{jk}},\sigma_{\mathrm{jk}}\right).$$

Separate, but identical models were fit for mass and wing length. Location‐ and species‐specific means ($\mu_{\mathrm{jk}}$) were modelled as normally distributed, while the standard deviations ($\sigma_{\mathrm{jk}}$) were modelled as half‐normal (normal distribution truncated to only positive values),

(2) $$\mu_{\mathrm{jk}}\sim N\left({\mu_{\text{mean}}}_{k},{\sigma_{\text{mean}}}_{k}\right)$$

$$\sigma_{\mathrm{jk}}\sim \mathrm{HN}\left({\mu_{\mathrm{SD}}}_{k},{\sigma_{\mathrm{SD}}}_{k}\right),$$

where ${\mu_{\text{mean}}}_{k}$ and ${\mu_{\mathrm{SD}}}_{k}$ represent the species‐specific means and ${\sigma_{\text{mean}}}_{k}$ and ${\sigma_{\mathrm{SD}}}_{k}$ represent the standard deviations. These parameters were themselves modelled as normally distributed, while standard deviations were half‐normal:

(3) $${\mu_{\text{mean}}}_{k}\sim N\left(\gamma_{\mu_{\text{mean}}},\phi_{\mu_{\text{mean}}}\right)$$

$${\sigma_{\text{mean}}}_{k}\sim \mathrm{HN}\left(\gamma_{\sigma_{\text{mean}}},\phi_{\sigma_{\text{mean}}}\right)$$

$${\mu_{\mathrm{SD}}}_{k}\sim \mathrm{HN}\left(\gamma_{\mu_{\mathrm{SD}}},\phi_{\mu_{\mathrm{SD}}}\right)$$

$${\sigma_{\mathrm{SD}}}_{k}\sim \mathrm{HN}\left(\gamma_{\sigma_{\mathrm{SD}}},\phi_{\sigma_{\mathrm{SD}}}\right).$$

For each trait, we used the estimated mean and standard deviation for each location‐species combination ($\mu_{\mathrm{jk}}$ and $\sigma_{\mathrm{jk}}$, respectively) to derive the CV for each location j, and species k as:

(4) $${\mathrm{CV}}_{\mathrm{jk}}=\frac{\sigma_{\mathrm{jk}}\;}{\mu_{\mathrm{jk}}},$$

to propagate uncertainty from estimates for σ and μ to estimates of CV. This approach explicitly takes into account the number of individuals captured for each station and species, avoiding known issues related to sample size when calculating CVs (Fluck et al. 2024; Yang et al. 2020).

We fit these models in a Bayesian framework, using the package *nimble* to interface with the NIMBLE software (de Valpine et al. 2017) via R (R Core Team 2025). We ran four chains for 50,000 iterations, with a burn‐in of 20,000, and a thinning rate of 20. Weakly informative priors were assigned for all parameters. All $\hat{R}$ values were ≤ 1.05, and the number of effective samples was > 400. For each location‐species estimate of CV, we extracted the posterior mean, $\hat{{\mathrm{CV}}_{\mathrm{jk}}}$, and the posterior standard deviation, $\hat{\tau_{\mathrm{jk}}}$, as well as species‐specific estimates, averaging the posteriors for the estimated CV of each species across all the locations, obtaining $\hat{{{\mathrm{CV}}_{\mathrm{SP}}}_{k}}$ and $\hat{{\tau_{\mathrm{SP}}}_{k}}$ (Figure 2B). We used these estimates in downstream models to test our hypotheses regarding the impact of various factors on within‐population phenotypic variation.

### 2.5 Quantifying the Drivers of Variation Across Species' Ranges

To assess the effect of the above mechanisms on phenotypic variation within each species, we used the location‐ and species‐specific estimates of CV, while accounting for the uncertainty in these estimates using an observation model,

(5) $$\hat{{\mathrm{CV}}_{\mathrm{jk}}}\sim N\left(\theta_{\mathrm{jk}},\hat{\tau_{\mathrm{jk}}}\right),$$

where $\hat{{\mathrm{CV}}_{\mathrm{jk}}}$ and $\hat{\tau_{\mathrm{jk}}}$ are the posterior mean and standard deviation derived from estimates of parameter ${\mathrm{CV}}_{\mathrm{jk}}$ (Equation 4) and $\theta_{\mathrm{jk}}$ represents the latent true state of CV. Both $\hat{{\mathrm{CV}}_{\mathrm{jk}}}$ and $\hat{\tau_{\mathrm{jk}}}$ were multiplied by 1000, to avoid small estimated values which can result in inefficient sampling and computational difficulties. All reported parameter estimates can be interpreted as the degree of change in (CV×1000), given a one unit change in a given covariate. As highlighted above, some covariates were logged—associated effect sizes should be interpreted accordingly. Parameter $\theta_{\mathrm{jk}}$ was modelled as normally distributed with mean $\mu_{\theta_{\mathrm{jk}}}$ and process error $\sigma_{\theta }$. The mean $\mu_{\theta_{\mathrm{jk}}}$ was modelled as a function of latitude, distance to range edge, spatial environmental variability, and temporal environmental variability, as:

(6) $$\theta_{\mathrm{jk}}\sim N\left(\mu_{\theta_{\mathrm{jk}}},\sigma_{\theta }\right)$$

$$\begin{array}{c} \mu_{\theta_{\mathrm{jk}}}=\alpha_{k}+{\beta_{1}}_{k}\times {\mathrm{Lat}}_{\mathrm{jk}}+{\beta_{2}}_{k}\times {\text{DistEdge}}_{\mathrm{jk}}+{\beta_{3}}_{k}\times {\text{SpatVar}}_{\mathrm{jk}}\\ +{\beta_{4}}_{k}\times {\text{TempVar}}_{\mathrm{jk}} \end{array}$$

$$\left[\begin{array}{c} \begin{array}{c} \alpha_{k}\\ {\beta_{1}}_{k} \end{array}\\ {\beta_{2}}_{k}\\ \;{\beta_{3}}_{k}\\ {\beta_{4}}_{k} \end{array}\right]\sim \mathrm{MVN}\left(\left[\begin{array}{c} \mu_{\alpha }\\ \mu_{\beta_{1}}\\ \mu_{\beta_{2}}\\ \mu_{\beta_{3}}\\ \mu_{\beta_{4}} \end{array}\right],\sum_{\theta }\right),$$

where α is the species‐specific intercept, the β parameters represent the species‐specific effect of the predictors on within‐population variation, and $\sum_{\theta }$ is a covariance matrix (5 × 5 matrix). Parameters $\mu_{\beta_{1}}$, $\mu_{\beta_{2}}$, $\mu_{\beta_{3}}$, $\mu_{\beta_{4}}$ represent the cross‐species effects of each covariate. We centered all variables by subtracting the mean of each predictor within each species. This allows for easier interpretation of the intercept and aids in model convergence. We calculated Variance Inflation Factors (VIF) (Zuur et al. 2010) to ensure no collinearity existed among the variables. All VIF < 1.7.

### 2.6 Quantifying the Drivers of Within‐Population Variation Among Species

To characterise how different species‐specific factors drive differences in within‐population variation among species, we modelled the species‐specific CV, $\hat{C{V_{\mathrm{SP}}}_{k}}$, using a normal distribution. As in (Equation 5), we account for the uncertainty in this metric using an observation model:

(7) $$\hat{{{\mathrm{CV}}_{\mathrm{SP}}}_{k}}\sim N\left(\xi_{k},\hat{{\tau_{\mathrm{SP}}}_{k}}\right),$$

where $\hat{{\tau_{\mathrm{SP}}}_{k}}$ represents the posterior standard deviation and $\hat{C{V_{\mathrm{SP}}}_{k}}$ represents the posterior mean. The mean, $\xi_{k}$ was modelled with a normal distribution, as a linear function of the species‐specific variables:

(8) $$\xi_{k}\sim N\left(\mu_{\xi_{k}},\sigma_{\xi }\right)$$

$$\begin{array}{c} \mu_{\xi_{k}}=\kappa +\eta_{k}+\zeta_{1}\times {\text{GenTime}}_{k}+\zeta_{2}\times {\mathrm{HWI}}_{k}+\zeta_{3}\times {\text{RangeSize}}_{\mathrm{jk}}\\ \;+\zeta_{4}\times {\text{MigStatus}}_{k} \end{array}$$

$$\eta_{k}\sim \mathrm{MVN}\left(0,P\sigma_{\text{phylo}}\right),$$

where κ is the grand intercept, $\eta_{k}$ is the species‐specific phylogenetic intercept, $\zeta_{1}$, $\zeta_{2}$, $\zeta_{3}$, and $\zeta_{4}$ are the covariate effects, and $\sigma_{\xi }$ is the process error. Parameter $\eta_{k}$, was modelled as a zero centered multivariate normal, where P is the correlation matrix derived from the pairwise phylogenetic distances among the 99 species included in the study (calculated from phylogenetic trees from BirdTree (Jetz et al. 2012)), and $\sigma_{\text{phylo}}$ is the scaling parameter for the magnitude of the phylogenetic intercepts. We used packages *ape* (Paradis and Schliep 2019) and *phytools* (Revell 2012) for data processing. We centered and scaled covariates to improve the computational efficiency of the model and ensured no collinearity among variables existed (VIF < 1.2).

We fit both within and among species models using the package *cmdstanr* (Gabry et al. 2023) to interface with Stan (Carpenter et al. 2017) via R (R Core Team 2025). We ran four chains for 5000 iterations, and warm‐up of 2500 iterations. We used weakly informative priors for all parameters. Package *MCMCvis* (Youngflesh 2018) was used for data processing and visualisation of the posteriors. For all parameters, $\hat{R}$ values were ≤ 1.01, the number of effective samples was > 400, and no model had divergent transitions. We used graphical posterior predictive checks to measure the ability of the model to generate data that is similar to the data used to fit the model. Generated data closely matched the observed data which indicates no major model misspecifications (Figure S7).

For all models, we report the mean and 89% credible interval (CI) for each parameter of interest. While the choice of 89% CI is arbitrary, it allows us to quantify parameter uncertainty without suggesting that Bayesian credible intervals are equivalent to statistical significance tests, as might be inferred from the conventional 95% CI interval (see McElreath 2018). We also report the probability that a given parameter is positive (calculated as the proportion of the posterior values that is > 0) as $p\left(\text{PARAMETER}>0\right)$. No units are reported as the CV, and the effect sizes derived from these data, are unitless.

## 3 Results

**FIGURE 3:**
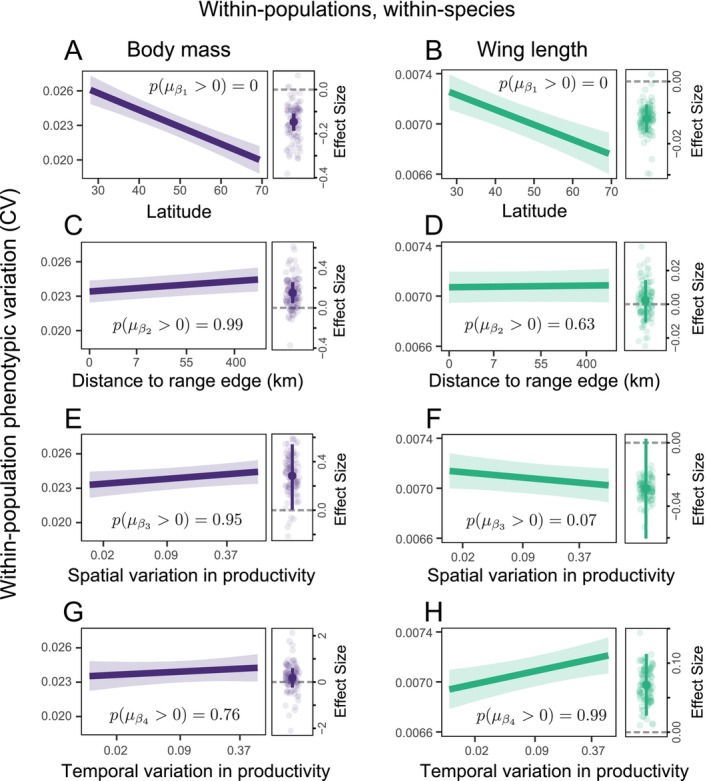
Multiple mechanisms drive within‐population phenotypic variation across species' ranges. Each set of plots in (A–H) represent the average effect of (A/B) latitude, (C/D) distance to range edge, (E/F) spatial variation in productivity, and (G/H) temporal variation in productivity on within‐population phenotypic variation (CV) for body mass (left column) and wing length (right column). In the plots at left of each set, bold lines represent the posterior means while ribbons represent the 89% credible intervals for the cross‐species effect, estimated as the mean of the species‐specific effects in the hierarchical model (Equation 6). In the plots at right of each set, bold points represent the posterior means of the effect of a given covariate, lines represent 89% credible intervals, while transparent jittered dots represent the species‐specific estimates (*N* = 99 in each case). Values of $p\left(\text{PARAMETER}>0\right)$ show the probability that a given parameter is positive (calculated as the proportion of the posterior values that is > 0).

We found strong evidence of a negative effect of latitude on the variation of both body mass ($\mu_{\beta_{1}}$ [Equation 6] = −0.147, 89% CI: [−0.188, −0.108], $p\left(\mu_{\beta_{1}}>0\right)$ = 0; Figure 3A; Figure S1A) and wing length ($\mu_{\beta_{1}}$ [Equation 6] = −0.012, 89% CI: [−0.016, −0.007], $p\left(\mu_{\beta_{1}}>0\right)$ = 0; Figure 3B; Figure S1B). We found strong evidence that populations located at the margins of species ranges have lower phenotypic variation when considering body mass ($\mu_{\beta_{2}}$ [Equation 6] = 0.150, 89% CI: [0.049, 0.253], $p\left(\mu_{\beta_{2}}>0\right)$ = 0.99; Figure 3A; Figure S3A), but not wing length ($\mu_{\beta_{2}}$ [Equation 6] = 0.002, 89% CI: [−0.011, 0.014], $p\left(\mu_{\beta_{2}}>0\right)$ = 0.63; Figure 3B; Figure S3B). Our results show some evidence that increased spatial variation in environmental conditions is associated with greater phenotypic variation for body mass ($\mu_{\beta_{3}}$ [Equation 6] = 0.280, 89% CI: [0.002, 0.549], $p\left(\mu_{\beta_{3}}>0\right)$ = 0.95; Figure 3A; Figure S4A) and with smaller variation in wing length ($\mu_{\beta_{3}}$ [Equation 6] = −0.029, 89% CI: [−0.060, 0.003], $p\left(\mu_{\beta_{3}}>0\right)$ = 0.07; Figure 3B; Figure S4B). Lastly, we found strong evidence that increased temporal variation in productivity is related to increased variation in wing length ($\mu_{\beta_{4}}$ [Equation 6] = 0.069, 89% CI: [0.024, 0.114], $p\left(\mu_{\beta_{4}}>0\right)$ = 0.99; Figure 3B; Figure S5A), but less evidence when considering body mass ($\mu_{\beta_{4}}$ [Equation 6] = 0.188, 89% CI: [−0.242, 0.624], $p\left(\mu_{\beta_{4}}>0\right)$ = 0.76; Figure 3A; Figure S5B).

**FIGURE 4:**
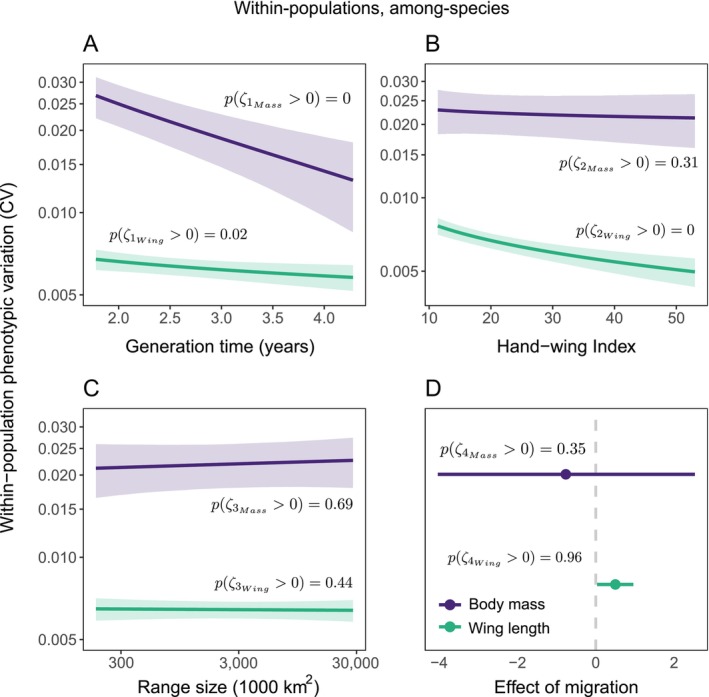
Within‐population phenotypic variation is mediated by species‐specific traits. Predicted within‐population phenotypic variation (CV) among species is shown according to (A) pace of life, measured as generation time, (B) dispersal ability, measured by the hand‐wing index; (C) range size; and (D) migratory status. Solid lines and points represent the posterior means (Equation 8), while ribbons (A–C) and horizontal lines (D) represent the 89% credible intervals. Values of $p\left(\text{PARAMETER}>0\right)$ show the probability that a given parameter is positive (calculated as the proportion of the posterior values that is > 0).

We found strong evidence of a negative effect of generation time on variation in both body mass ($\zeta_{1}$ [Equation 8] = −2.554, 89% CI: [−3.402, −1.689], $p\left(\zeta_{1}>0\right)$ = 0; Figure 4A) and wing length ($\zeta_{1}$ [Equation 8] = −0.178, 89% CI: [−0.320, −0.035], $p\left(\zeta_{1}>0\right)$ = 0.02; Figure 4A). Natal dispersal had a negative effect on variation in wing length ($\zeta_{2}$ [Equation 8] = −0.441, 89% CI: [−0.580, −0.301], $p\left(\zeta_{2}>0\right)$ = 0; Figure 4B), but no effect on variation in body mass ($\zeta_{2}$ [Equation 8] = −0.273, 89% CI: [−1.187, 0.632], $p\left(\zeta_{2}>0\right)$ = 0.31; Figure 4B). We found little support for the effect of geographic range size on variation for both body mass ($\zeta_{3}$ [Equation 8] = 0.261, 89% CI: [−0.615, 1.126], $p\left(\zeta_{3}>0\right)$ = 0.69; Figure 4C) and wing length ($\zeta_{3}$ [Equation 8] = −0.014, 89% CI: [−0.152, 0.121], $p\left(\zeta_{3}>0\right)$ = 0.44; Figure 4C). Our results support the notion that migratory species generally have larger phenotypic variation in wing length ($\zeta_{4}$ [Equation 8] = 0.500, 89% CI: [0.034, 0.959], $p\left(\zeta_{4}>0\right)$ = 0.96; Figure 4D), but not when considering body mass ($\zeta_{4}$ [Equation 8] = −0.764, 89% CI: [−4.034, 2.531], $p\left(\zeta_{4}>0\right)$ = 0.35; Figure 4D).

## 4 Discussion

Using a hierarchical Bayesian approach to leverage individual‐level data from more than 197,000 individuals from 99 bird species, we assessed the degree to which geographic, environmental, and life history processes shape observed within‐population phenotypic variation (i.e., variation among individuals). Results highlight the importance of multiple, non‐mutually exclusive mechanisms in driving these patterns of biodiversity as well as the considerable variation in within‐population phenotypic variation observed both across species' ranges and among species.

On average, the degree of phenotypic variation (i.e., variation among individuals within populations) varied by 107% and 38% across species' ranges, for body size (Figure S6A) and wing length (Figure S6B), respectively. That is, some populations exhibited variation in body mass more than double that of other populations within the same species. The degree of variation also varied considerably among species, with the golden‐crowned kinglet ( *Regulus satrapa* ) exhibiting three times greater variation in (within‐population) body mass than the species with the lowest degree of variation, the brown thrasher ( *Toxostoma rufum* ) (Figure S6C). For wing length, the Pacific wren ( *Troglodytes pacificus* ) exhibited 1.7 times greater variation than the yellow‐bellied sapsucker ( *Sphyrapicus varius* ) (Figure S6D).

### 4.1 Phenotypic Variation Varies Predictably Across Species' Ranges

Latitude plays a key role in structuring various dimensions of biodiversity (Stevens and Tello 2018). Results show that phenotypic variation within populations is no exception—on average, there was a 6.6% and 1.7% decrease in variation with a 10‐degree increase in latitude (31% and 7.3% decrease across the entire latitudinal range) for body mass and wing length, respectively (Figure 3 and Figure S2). From a mechanistic perspective, we attribute the decrease in phenotypic variation at higher latitudes to a serial founder effect in the establishment of more northerly populations, which were established more recently. As ice sheets retreated northward after the Last Glacial Maximum approximately 20,000 years ago (Hewitt 2000), bird populations (re)colonised these regions. The series of successive sampling events to establish these populations would be expected to result in decreased phenotypic variation (Kolbe et al. 2012; Mayr 1942). This gradient in variation is consistent with genetic evidence from multiple taxa (Adams and Hadly 2013; Miraldo et al. 2016), including birds (Smith et al. 2017), where lower intraspecific genetic variation has generally been observed at higher latitudes.

The position of a given population within a species range might also be expected to structure phenotypic variation. Populations near range margins typically experience reduced gene flow (Eckert et al. 2008; Kirkpatrick and Barton 1997; Sexton et al. 2009) and are typically characterized by lower abundances (Brown 1984), which has been linked to lower phenotypic variation (Agnew 1968; Eckert et al. 2008). Local adaptation to specific suboptimal environmental conditions at range margins may also reduce phenotypic variation (Hoffmann and Blows 1994), which can be associated with lower fitness (Bontrager and Angert 2019) and higher local extinction risk (Maurer and Taper 2002). Results support this notion when considering body mass—there was a 4.5% increase in variation across the entire range of observed distances—but not when considering wing length. This trait‐specific response may be due to the differential constraints on these two traits. For instance, wing length may be constrained by flight‐related requirements.

Various pieces of evidence suggest that both spatial and temporal variation in environmental conditions may shape phenotypic variation. Spatially heterogeneous environments provide a wide range of ecological niches, potentially reducing competition, minimizing trait overlap, and diversifying food resources (Vernham et al. 2023), which are thought to facilitate increased intraspecific phenotypic variation (Cordero and Epps 2012). Results provide some evidence for this notion considering body mass, with a 5.0% increase in variation across the range of observed spatial variation (Figure 3), though there was some uncertainty regarding this estimated effect. We used estimates of annual productivity as our environmental metric as this is the manifestation of a number of different environmental conditions, and likely plays an important role in the availability of food resources for these species (Cody 1981; Read et al. 2020). The relationship between phenotypic variation and other environmental variables, however, may differ. Findings for variation in wing length, were contrary to our expectations, decreasing as spatial variation in environmental conditions increased, though some uncertainty was present. Additional factors, such as the quality of the environment for a given species at a given location, might also play a role in driving phenotypic variation. In high‐quality environments, trait values may converge toward an optimum, reducing variability (Teder et al. 2008). This could potentially contribute to the contrasting results if this effect varies among traits.

Temporal environmental fluctuations are linked to fluctuating selection pressures (Siepielski et al. 2009, 2017), which might be expected to result in increased genetic and phenotypic diversity (Yamamichi et al. 2023). Under fluctuating conditions, the optimal phenotype in a population might vary temporally. At any given point in time, a population might therefore consist of individuals best suited for a range of environmental conditions that are observed periodically in that environment. Temporal variation in environmental conditions is known to drive diverse responses in wild populations (Bernhardt et al. 2020), impacting genetic composition (Bradshaw and Holzapfel 2001; Gienapp et al. 2008), morphology (Anderson et al. 2019; Garant et al. 2004; Pergams and Lawler 2009; Youngflesh et al. 2022), phenology (Charmantier et al. 2006; Réale et al. 2003; Youngflesh et al. 2021), geographic ranges (Chen et al. 2011), and life history traits (Youngflesh et al. 2025), all of which might themselves influence phenotypic variation. We found strong evidence that greater temporal variation in productivity is associated with increased variation in wing length, with a 4.4% increase in variation across the range of observed temporal variation, though little evidence when considering body mass (Figure 3). If different wing lengths (but not different body masses) are advantageous under different environmental conditions, potentially due to the importance of foraging efficiency, more phenotypic variation in this trait might be expected in these more variable environments.

### 4.2 Life History Predicts the Magnitude of Intraspecific Phenotypic Variation

Pace‐of‐life, the position along the slow‐fast continuum that species fall, plays a critical role in how species interact with their environments (Healy et al. 2019; Youngflesh et al. 2025). Among species, we found strong evidence that fast‐paced species exhibit greater within‐population phenotypic variation, considering both body mass and wing length, providing large‐scale empirical support for this long‐standing theoretical assertion (Schultz 1989). Species near the fast end of the continuum exhibited variation in body mass that was 104.1% larger than species near the slow end of the continuum (i.e., nearly double), 16.5% larger for wing length. The higher reproductive rates exhibited by fast‐paced species (typically accompanied by shorter generation times) are thought to be the mechanism driving this association. With more offspring per generation, there is an increased potential for genetic and phenotypic variation, all else equal (Schultz 1989; Wright et al. 2019).

Given the important role that juvenile dispersal plays in gene flow and population dynamics (Bowler and Benton 2005; Burns and Broders 2014; Ronce 2007), we expected species with higher dispersal capacities to exhibit greater phenotypic variation within populations, as more mixing would occur among populations in these cases. Results, however, suggest the opposite for wing length—variation in wing length was 54% higher for species with lower dispersal ability compared to species with higher dispersal ability—with no strong evidence for the effect of body mass. These results are however consistent with previous findings suggesting that high natal dispersal can decrease diversification rates by limiting the effectiveness of geographic barriers to gene flow, resulting in a homogenization effect (Claramunt et al. 2012; Weeks and Claramunt 2014). In this case, fewer differences among populations could result in lower variation within populations, even if more exchange of individuals was occurring. Selection pressures acting on dispersing individuals may also favour particular wing morphologies for efficient flight, which could lead to reduced variation within species with higher dispersal abilities (Burns and Broders 2014).

Contrary to our expectations, phenotypic variation in both body mass and wing length appeared largely unrelated to species geographic range size. Previous studies have suggested that species with larger range sizes experience more diverse habitat conditions (Hawkins and Felizola Diniz‐Filho 2006; Li et al. 2016; Pohlman et al. 2005; Slatyer et al. 2013), which might result in higher variation across a species range and potentially more variation within a population, conditional on sufficient dispersal of these phenotypes across space. Prior work has also shown larger range sizes to be associated with higher genetic variation (Leffler et al. 2012). The lack of evidence for this hypothesized mechanism may be due to limited gene flow across species ranges, decreasing the degree of observed variation in these traits at the population level, and/or from stabilizing selection acting at the population level (Pélabon et al. 2010).

Regarding the effect of migratory behaviour, we show evidence that migratory species generally have larger phenotypic variation when considering wing length (4.7% larger), but not body mass. While seasonally migratory species experience diverse environmental pressures and need to cope with different ecological conditions throughout their annual cycle (Avgar et al. 2014; Webster et al. 2002), there may be local adaptation to specific conditions experienced on the breeding or non‐breeding grounds (Hedenström 2008; Wanamaker et al. 2020). For many migratory species, individuals from a single breeding population may spend the non‐breeding season at different locations (Cohen et al. 2018), which can promote mixing of individuals that might be adapted to different non‐breeding conditions (Finch et al. 2017). This could potentially result in larger phenotypic variation in these populations. However, other factors related to migration, beyond simply migratory status (migrant vs. non‐migrant), such as the degree of migratory connectivity (Cohen et al. 2018) and the importance of phenotypic traits for persisting in non‐breeding compared to breeding locations (Norris 2005), likely play an important role in shaping this variation. For instance, species that exhibit weak connectivity between the breeding and non‐breeding grounds (i.e., individuals from a single breeding site disperse across multiple non‐breeding sites), will likely have greater phenotypic variation compared to species with strong migratory connectivity, all else equal (Webster et al. 2002). Unfortunately, we lack a comprehensive understanding of connectivity across the full annual cycle for most migratory bird species, making it difficult to assess the role that these dynamics play. Additional factors, such as partial migration where some subset of individuals or populations of a species migrate while others do not, add additional complexities to understanding the role of migration on phenotypic variation.

### 4.3 Non‐Mutually Exclusive Processes Shape Phenotypic Variation Across Scales

Our findings demonstrate that a number of non‐mutually exclusive processes act in concert to give rise to these pronounced differences in phenotypic variation. Some support exists for each of the four hypothesized drivers of phenotypic variation across species ranges, and for three of the four hypothesized drivers when comparing variation among species. While the absolute estimated variation in body mass, characterized by the CV, was about three times larger than the variation in wing length (Figure S6), this is expected given the allometric relationships between volume (of which body mass is a proxy) and length (of which wing length is a measure) (Lande 1977; Pélabon et al. 2020). That is, CV for a volumetric measure should be three times greater than for a linear measure. For several of the proposed mechanisms (i.e., distance to range edge, temporal environmental variation, HWI, migration status), support existed for one trait but not the other and in 1 of the 8 cases, the direction of support differed across traits. This suggests the way in which these processes impact phenotypic variation varies in a trait‐specific manner, reflecting distinct ecological and evolutionary constraints on different morphological traits (Murren et al. 2015). For example, while wing length variation may be more constrained by flight efficiency and aerodynamics (Alerstam et al. 2007; Wright et al. 2016), body mass is likely more constrained by metabolic and energetic demands (Banavar et al. 2002).

While we focus specifically on variation among individuals within populations, variation among populations is another important component of intraspecific phenotypic variation (i.e., ITV). Average variation within populations (0.024 for body mass and 0.007 for wing length) was larger than average variation among populations within species (0.011 for body mass and 0.004 for wing length) though smaller than variation among species (0.208 for body mass and 0.051 for wing length). The mechanisms and consequences of these two dimensions of intraspecific phenotypic variation (within and among populations) are distinct and must be decoupled if we are to properly characterize how biodiversity varies across scales, including the role that this variation plays in observed patterns of occurrence and niche breadth (Violle et al. 2012). Some variation also exists within individuals, reflecting a combination of measurement error and true individual‐level change over time—fewer than 7% of individuals were captured more than three times across this study. While this within‐individual variation should not systematically bias the inference made in this study, further exploration of these patterns is warranted. We estimated the variance within individuals to be approximately 14% (mass) and 15% (wing length) of the total observed phenotypic variation for a given species.

We expect that these differences in phenotypic variation could vary across other axes as well. For instance, while we focus on male birds in this study, the response of female birds may differ as they have different physiological demands (and potentially constraints) imposed on them due to the requirements of reproduction. Similarly, the degree of phenotypic variation, both at the population and individual level, may be changing over time, in concert with changes in the mean of these phenotypic traits (Youngflesh et al. 2022). Other mechanisms, not addressed here such as interspecific competition, may also play a role in driving within‐population phenotypic variation. These points deserve additional consideration and should be the focus of future study.

### 4.4 Implications for Understanding Ecological Systems

Synthesising a collection of data, including individual‐level phenotypes, species‐level traits, and environmental conditions, our study demonstrates how within‐population variation differs substantially both within and among species and provides empirical evidence for several mechanisms hypothesized to shape this variation. Geographic, environmental, and life history processes act simultaneously to structure these patterns, with important implications for eco‐evolutionary dynamics. This variation might regulate a variety of ecological interactions (Palkovacs and Post 2009), including competition (Clark 2010) both within and among species, with implications for the composition of communities and ecosystems (Fussmann et al. 2007; Jung et al. 2010) as well as how they are likely to respond to ongoing global change (Des Roches et al. 2021). Intraspecific variation can impact not only the resilience of populations to perturbations (Forsman 2014) but also the degree to which these populations can adapt (Jump et al. 2009). Ultimately, phenotypic variation is the raw material upon which natural selection acts (Bolnick et al. 2003; Kingsolver and Pfennig 2007). Characterising the axes along which phenotypic variation varies and the processes that drive these patterns, is crucial if we are to predict which species and systems might be most susceptible to future change (Moran et al. 2016; Radchuk et al. 2019). While efforts to combat the homogenization of the biotic environment are often focused at the level of species, the importance of intraspecific variation, including within populations, must also be recognised (Des Roches et al. 2018).

## Author Contributions

C.Y. conceptualised the project, V.Z. and C.Y. developed the methods, analysed the data, and wrote the paper.

## Supporting information


**Figure S1:** ele70244‐sup‐0001‐FigureS1.pdf.

**Figure S2:** ele70244‐sup‐0002‐FigureS2.pdf.

**Figure S3:** ele70244‐sup‐0003‐FigureS3.pdf.

**Figure S4:** ele70244‐sup‐0004‐FigureS4.pdf.

**Figure S5:** ele70244‐sup‐0005‐FigureS5.pdf.

**Figure S6:** ele70244‐sup‐0006‐FigureS6.pdf.

**Figure S7:** ele70244‐sup‐0007‐FigureS7.pdf.

**Data S1:** ele70244‐sup‐0008‐DataS1.docx.

## Data Availability

Data from the Monitoring Avian Productivity and Survivorship (MAPS) program (DeSante et al. 2004) is curated and managed by The Institute for Bird Populations and was queried from the MAPS database on 2019‐10‐16. MAPS data necessary to fit the models presented here are archived on Zenodo (DOI: https://doi.org/10.5281/zenodo.17159488). All code to reproduce analyses is freely available on GitHub (https://github.com/vivizulian/WithinPopsPhenoVar/) and is archived on Zenodo (DOI: https://doi.org/10.5281/zenodo.17344600). Dynamic Habitat Index data are available from Radeloff et al. (2019). Bird range maps are available from (BirdLife International and Handbook of the Birds of the World 2022), bird trait data are available from the AVONET database (Tobias et al. 2022), bird generation time data are available from Bird et al. (2020), and bird phylogenetic data are available from birdtree.org (Jetz et al. 2012).
