# Supplementary figures and images for: Geography, Environmental Conditions and Life History Shape Patterns of Within‐Population Phenotypic Variation in North American Birds
Source: Ecol Lett. 2025 Nov 9;28(11):e70244. doi: 10.1111/ele.70244 (PMC12596938; doi:10.1111/ele.70244)

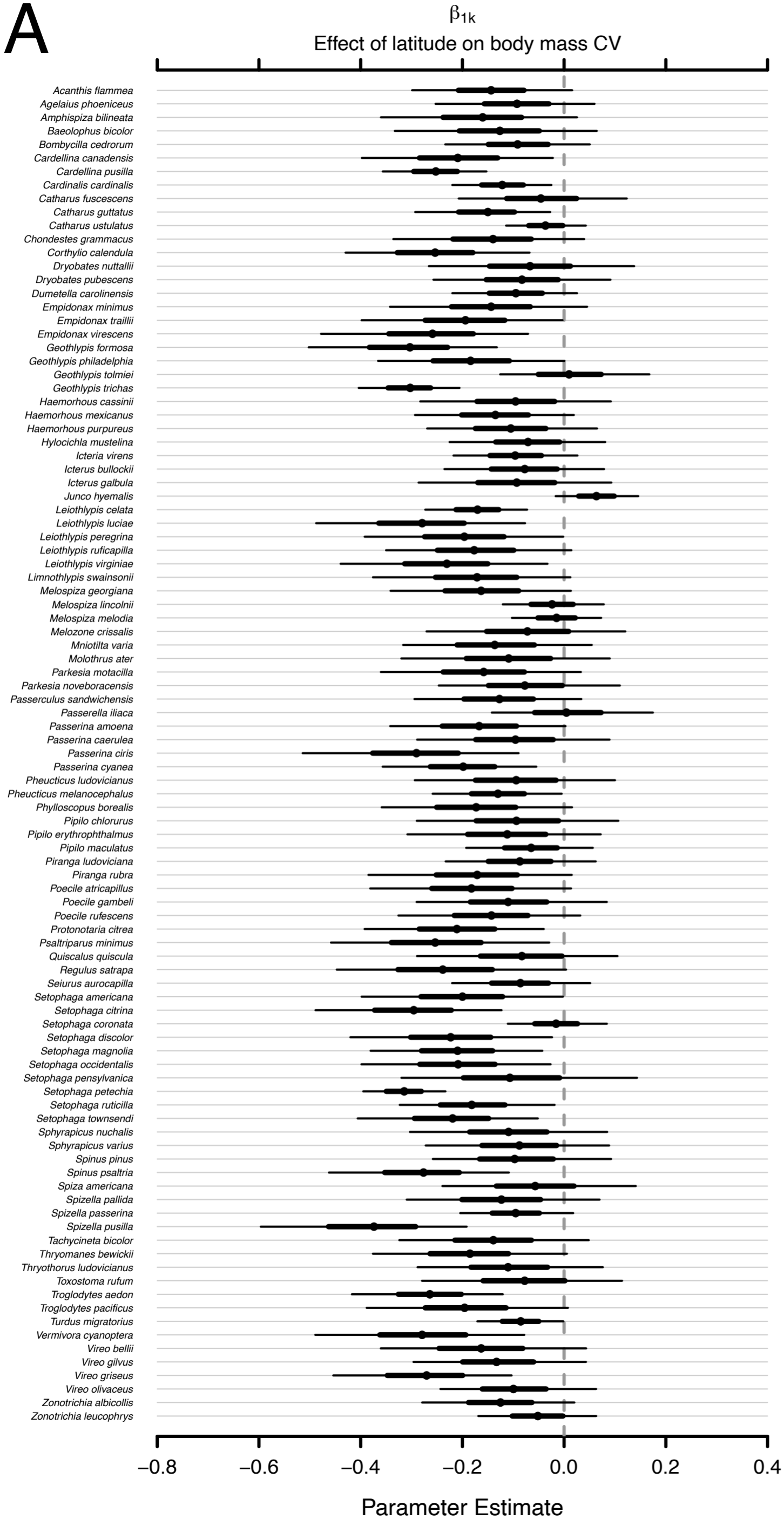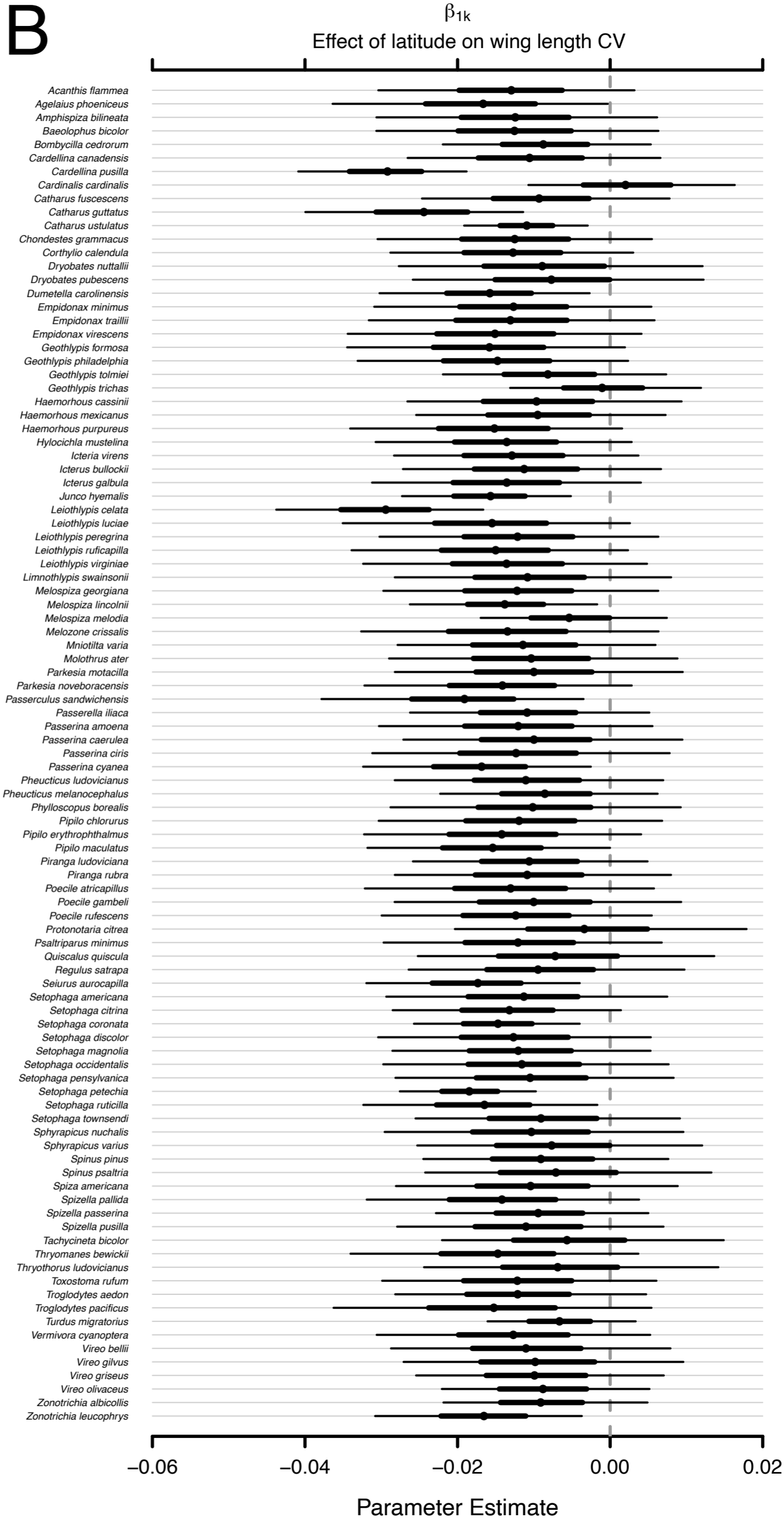

Supplement: Supplementary file 1 — Figure S1: ele70244‐sup‐0001‐FigureS1.pdf. [file ELE-28-0-s004.pdf]

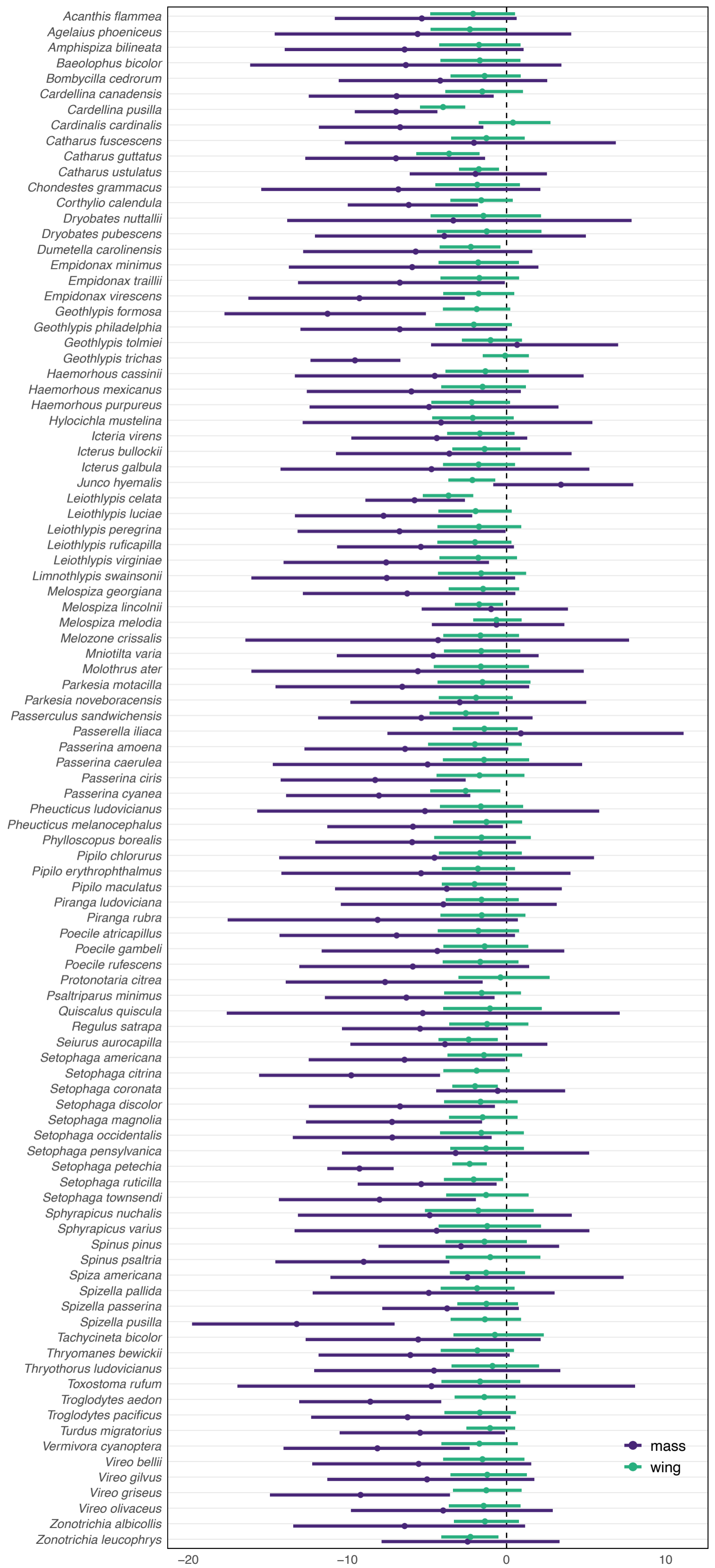

% Change on body mass and wing length CV per 10 degree latitude increase

Supplement: Supplementary file 2 — Figure S2: ele70244‐sup‐0002‐FigureS2.pdf. [file ELE-28-0-s003.pdf]

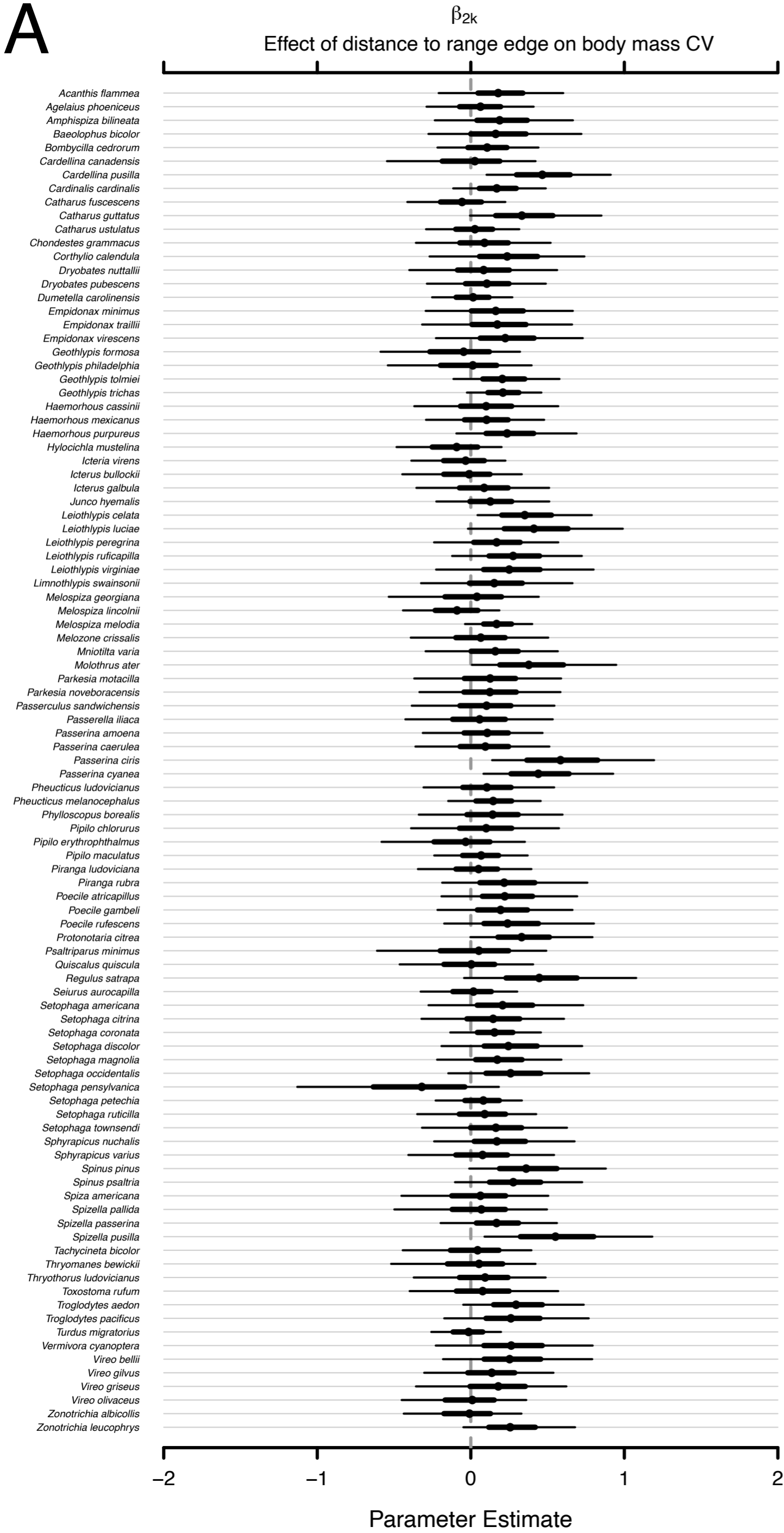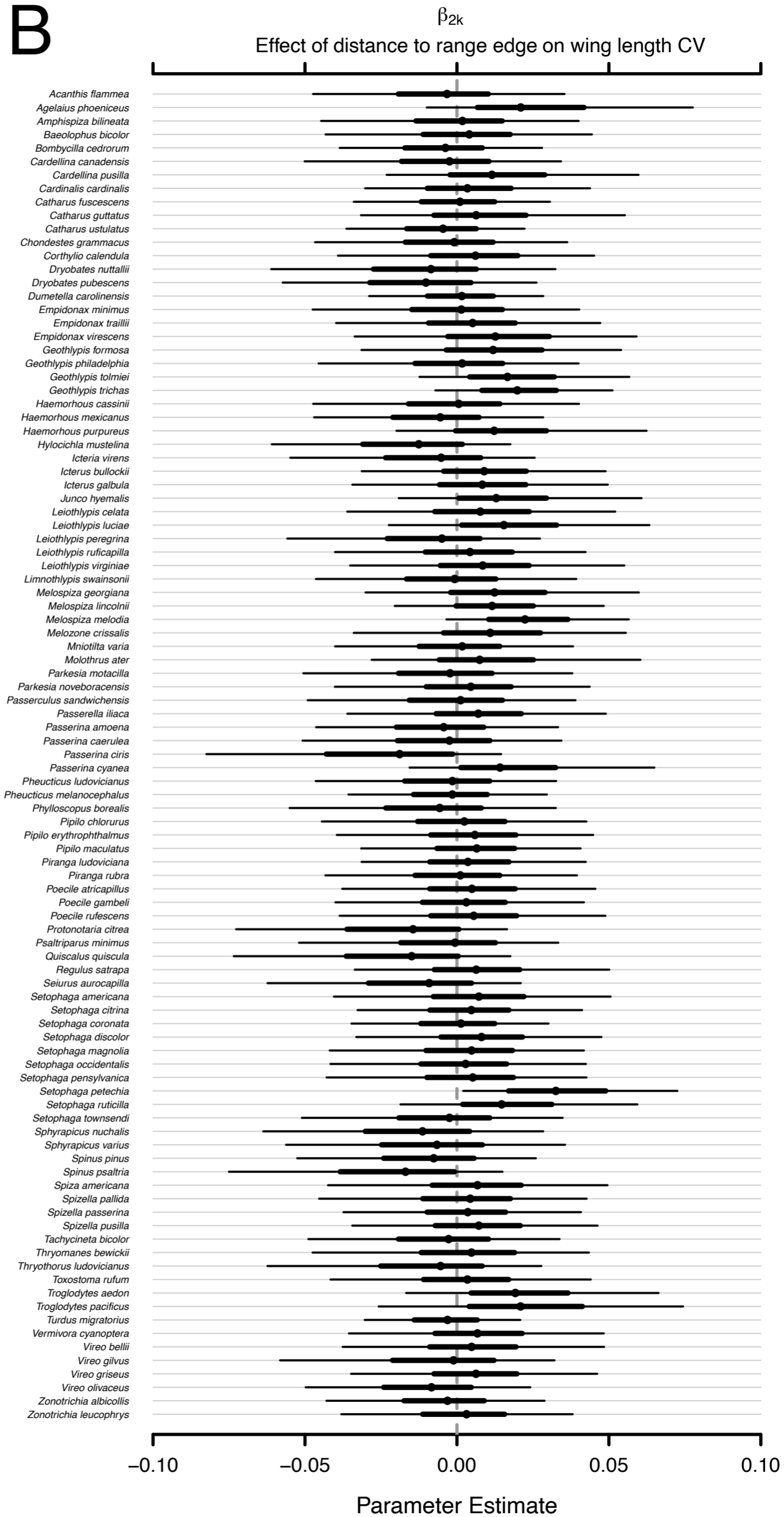

Supplement: Supplementary file 3 — Figure S3: ele70244‐sup‐0003‐FigureS3.pdf. [file ELE-28-0-s007.pdf]

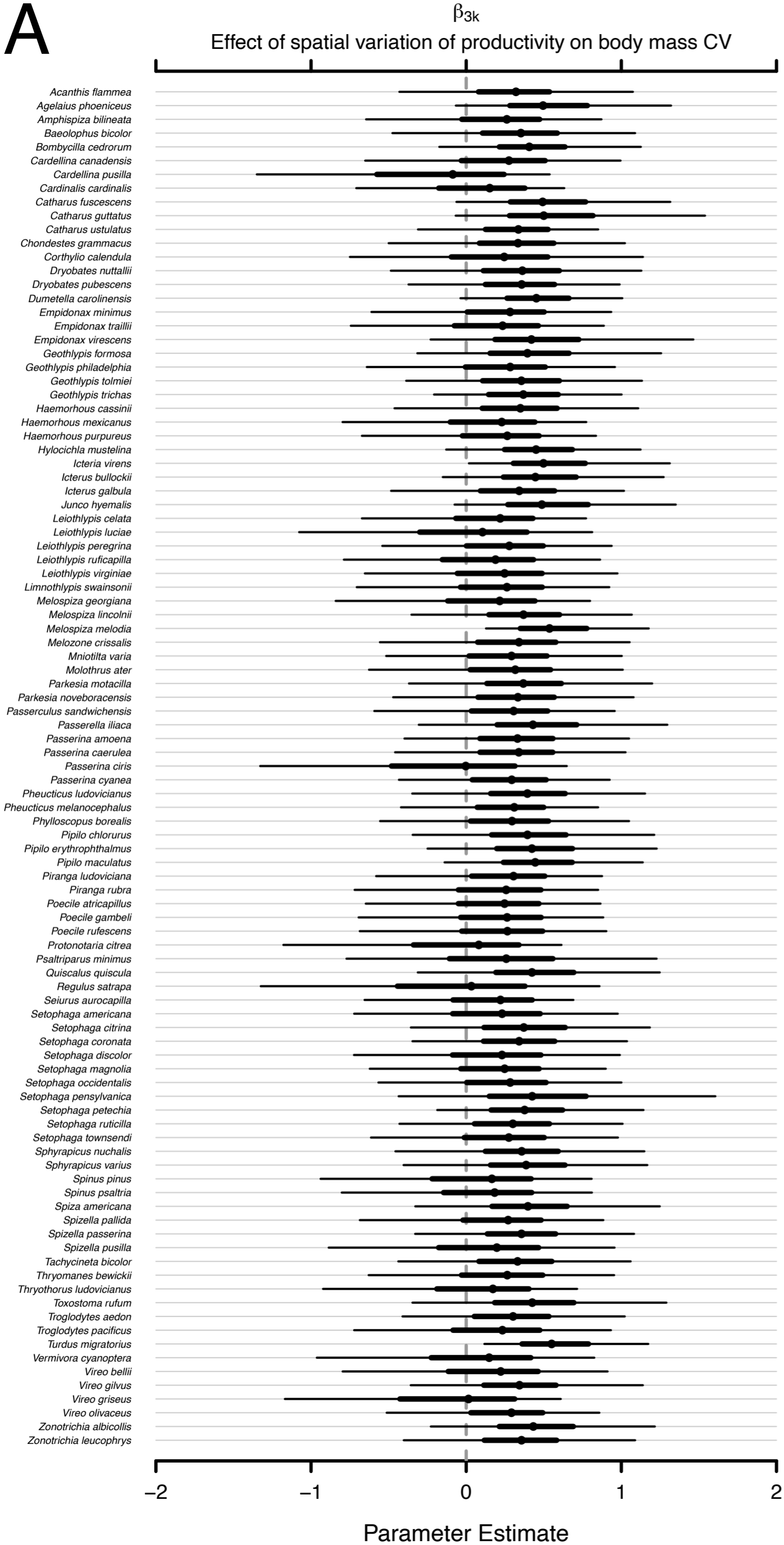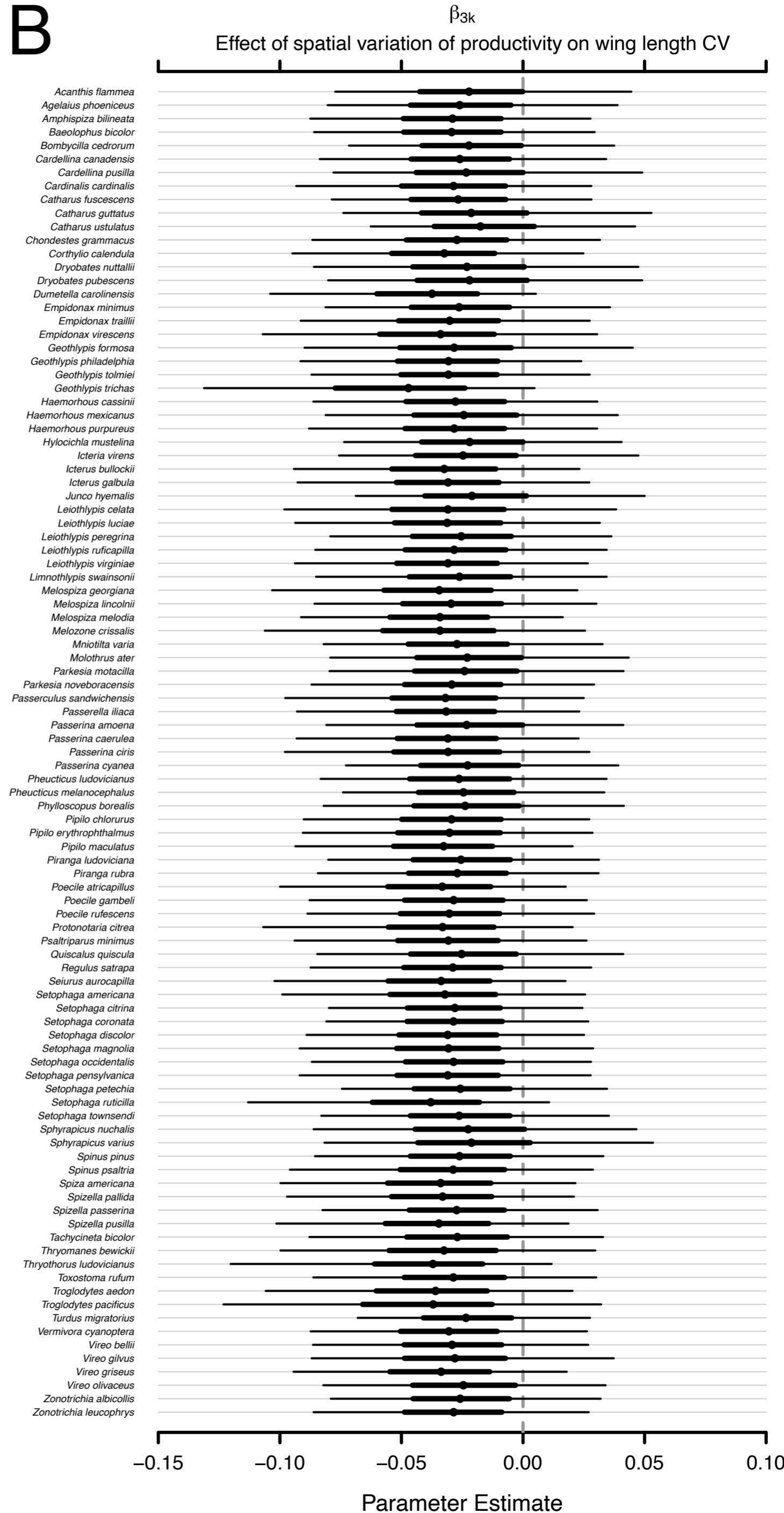

Supplement: Supplementary file 4 — Figure S4: ele70244‐sup‐0004‐FigureS4.pdf. [file ELE-28-0-s008.pdf]

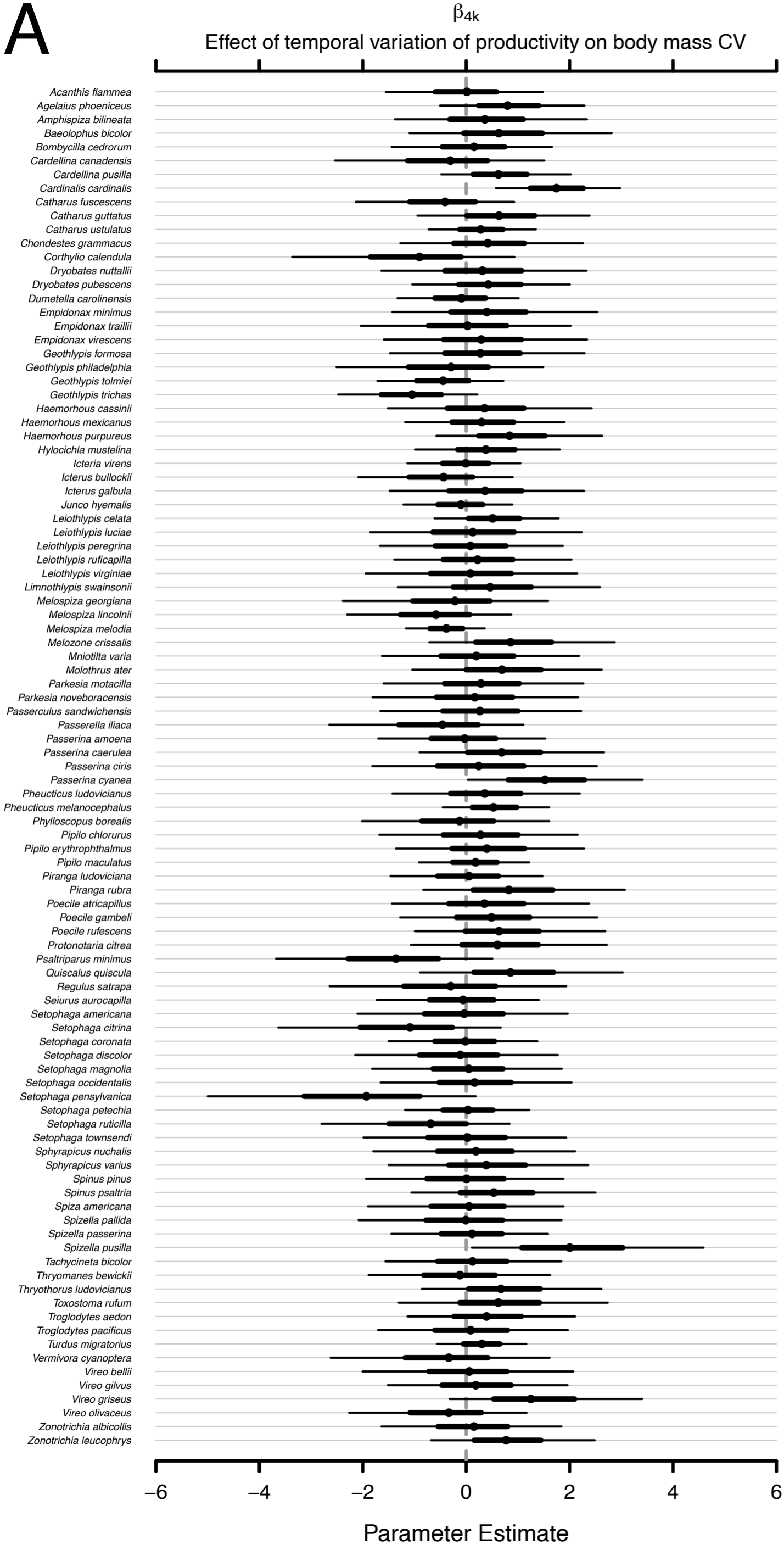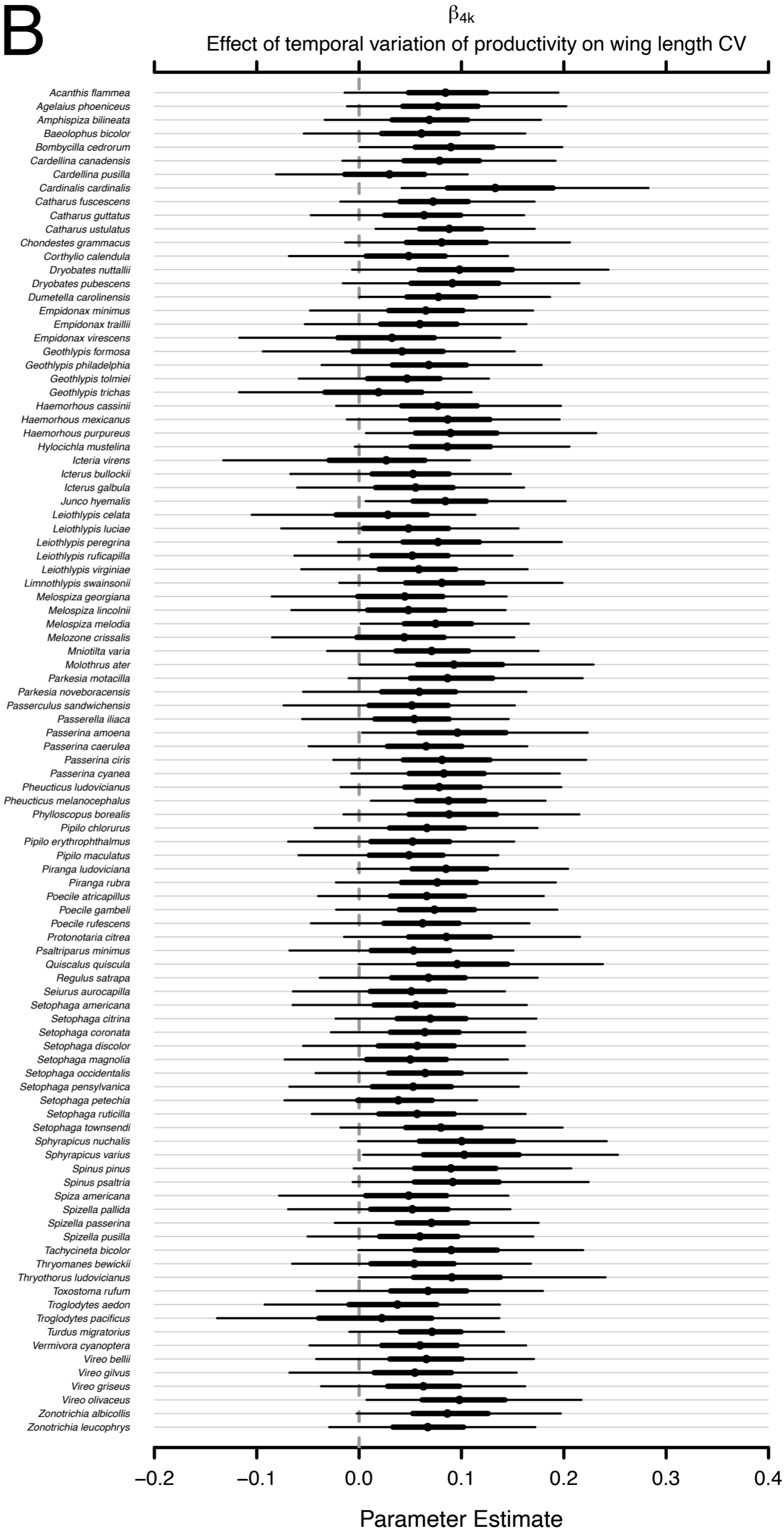

Supplement: Supplementary file 5 — Figure S5: ele70244‐sup‐0005‐FigureS5.pdf. [file ELE-28-0-s005.pdf]

**A** Body mass

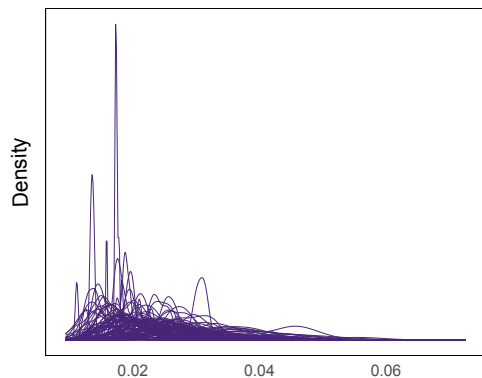

**B** Wing length

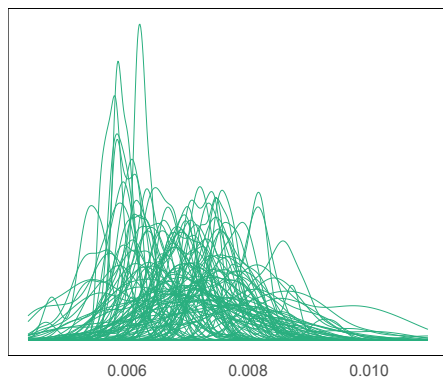

CV within populations

**C**

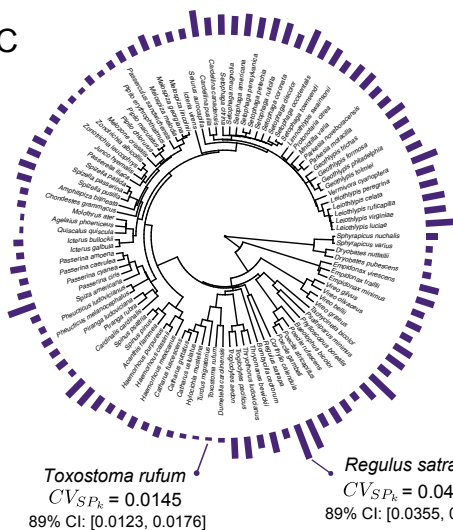

**D**

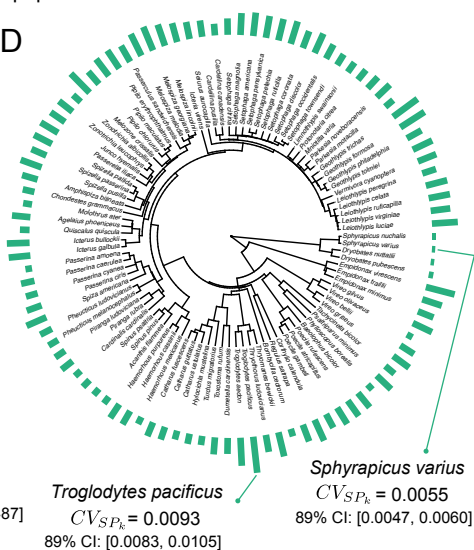

Supplement: Supplementary file 6 — Figure S6: ele70244‐sup‐0006‐FigureS6.pdf. [file ELE-28-0-s006.pdf]
