# Supplementary material for: Geography, Environmental Conditions and Life History Shape Patterns of Within‐Population Phenotypic Variation in North American Birds
Source: Ecol Lett. 2025 Nov 9;28(11):e70244. doi: 10.1111/ele.70244 (PMC12596938; doi:10.1111/ele.70244)
Supplement: Supplementary file 7 — Figure S7: ele70244‐sup‐0007‐FigureS7.pdf. [file ELE-28-0-s002.pdf]

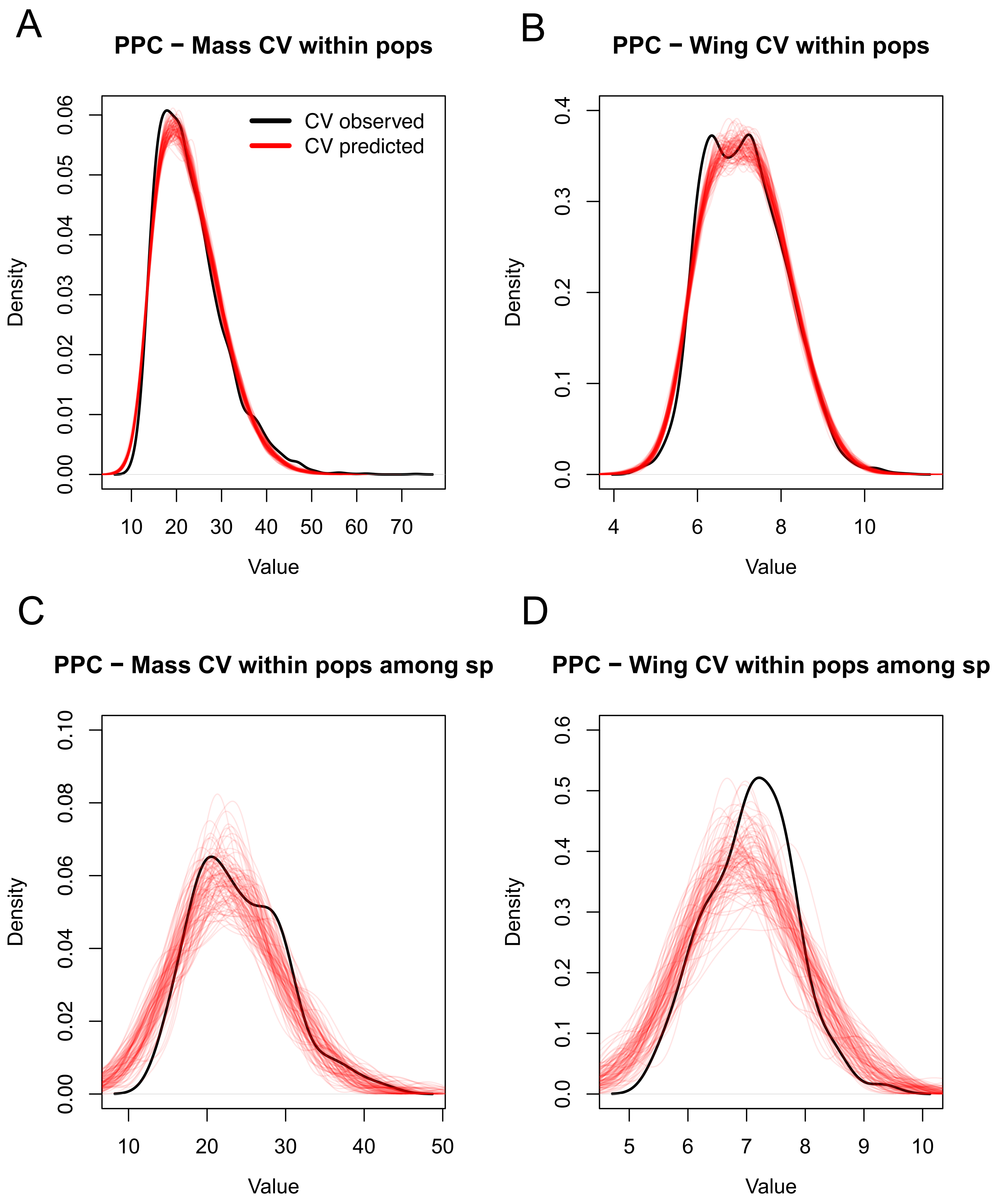
A
PPC − Mass CV within pops
CV observed
CV predicted
Density
0.00
0.01
0.02
0.03
0.04
0.05
0.06
10
20
30
40
50
60
70
Value
B
PPC − Wing CV within pops
Density
0.0
0.1
0.2
0.3
0.4
4
6
8
10
Value
C
PPC − Mass CV within pops among sp
Density
0.00
0.02
0.04
0.06
0.08
0.10
10
20
30
40
50
Value
D
PPC − Wing CV within pops among sp
Density
0.0
0.1
0.2
0.3
0.4
0.5
0.6
5
6
7
8
9
10
Value
