# Supplementary material for: Geography, Environmental Conditions and Life History Shape Patterns of Within‐Population Phenotypic Variation in North American Birds
Source: Ecol Lett. 2025 Nov 9;28(11):e70244. doi: 10.1111/ele.70244 (PMC12596938; doi:10.1111/ele.70244)
Supplement: Supplementary file 8 — Data S1: ele70244‐sup‐0008‐DataS1.docx. [file ELE-28-0-s001.docx]

**SUPPORTING INFORMATION**

**Figure S1:** **Posterior estimates for** $\boldsymbol{\beta}_{\boldsymbol{1}_{\boldsymbol{k}}}$ **[Eq. 6].** These denote the species-specific effect size of latitude on A) body mass and B) wing length CV. Points represent the posterior means, while thick and thin lines represent the 50% and 89% credible intervals, respectively. The vertical dashed line represents zero.

**Figure S2:** **Percentage change (**$\boldsymbol{\% change}$**) in body mass and wing length CV per 10-degree latitude increase.** The degree of variation is calculated using the predicted CVs at the maximum and minimum latitudes, while accounting for the extension of the range for each species. Results were multiplied by 10 to get the percentage change per 10-degree latitude. Dots represent the posterior mean change in CV, while the horizontal lines represent the 89% credible intervals. Percentage changes were calculated as $\left( \left( \frac{\left( \hat{{CV}_{k_{MAX}}}- \hat{{CV}_{k_{MIN}}} \right)/ \hat{{CV}_{k_{MIN}}}}{{Lat}_{k_{MAX}}- {Lat}_{k_{MIN}}} \right)*100 \right)*10$.

**Figure S3:** **Posterior estimates for** $\boldsymbol{\beta}_{\boldsymbol{2}_{\boldsymbol{k}}}$ **[Eq. 6].** These denote the species-specific effect size of distance to range edge on A) body mass and B) wing length CV. Points represent the posterior means, while thick and thin lines represent the 50% and 89% credible intervals, respectively. The vertical dashed line represents zero.

**Figure S4:** **Posterior estimates for** $\boldsymbol{\beta}_{\boldsymbol{3}_{\boldsymbol{k}}}$ **[Eq. 6].** These denote the species-specific effect size of spatial variation of productivity on A) body mass and B) wing length CV. Points represent the posterior means, while thick and thin lines represent the 50% and 89% credible intervals, respectively. The vertical dashed line represents zero.

**Figure S5:** **Posterior estimates for** $\boldsymbol{\beta}_{\boldsymbol{4}_{\boldsymbol{k}}}$ **[Eq. 6].** These denote the species-specific effect size of temporal variation of productivity on A) wing length and B) body mass CV. Points represent the posterior means, while thick and thin lines represent the 50% and 89% credible intervals, respectively. The vertical dashed line represents zero.

**Figure S6: The magnitude of intraspecific phenotypic variation differs both within and among species.** The degree of within-population phenotypic variation in A) body mass was about three times larger than the variation in B) wing length. The phylogeny shows the 99 species included in this study, with the bar height representing the average within-population phenotypic variation in C) body mass and D) wing length for each species. Pointers show the species with the greatest and the smallest within-population phenotypic variation, respectively, for body mass (*Regulus satrapa* and *Toxostoma rufum*) and wing length (*Troglodytes pacificus* and *Sphyrapicus varius*).

**Figure S7:** **Density plots for the observed response variable data (*CV observed*) and data simulated from the posterior predictive distribution (*CV predicted*).** These plots were used to ensure models were able to generate data that is similar to the data used to fit the model investigating how latitude, distance to range edge, spatial and temporal environmental variation drive differences in the variation in A) body mass and B) wing length across species range [Eqs. 5, 6]; and how generation time, hand wing index, range size and migratory status affect differences in variation in C) body mass and D) wing length among species [Eqs. 7, 8].


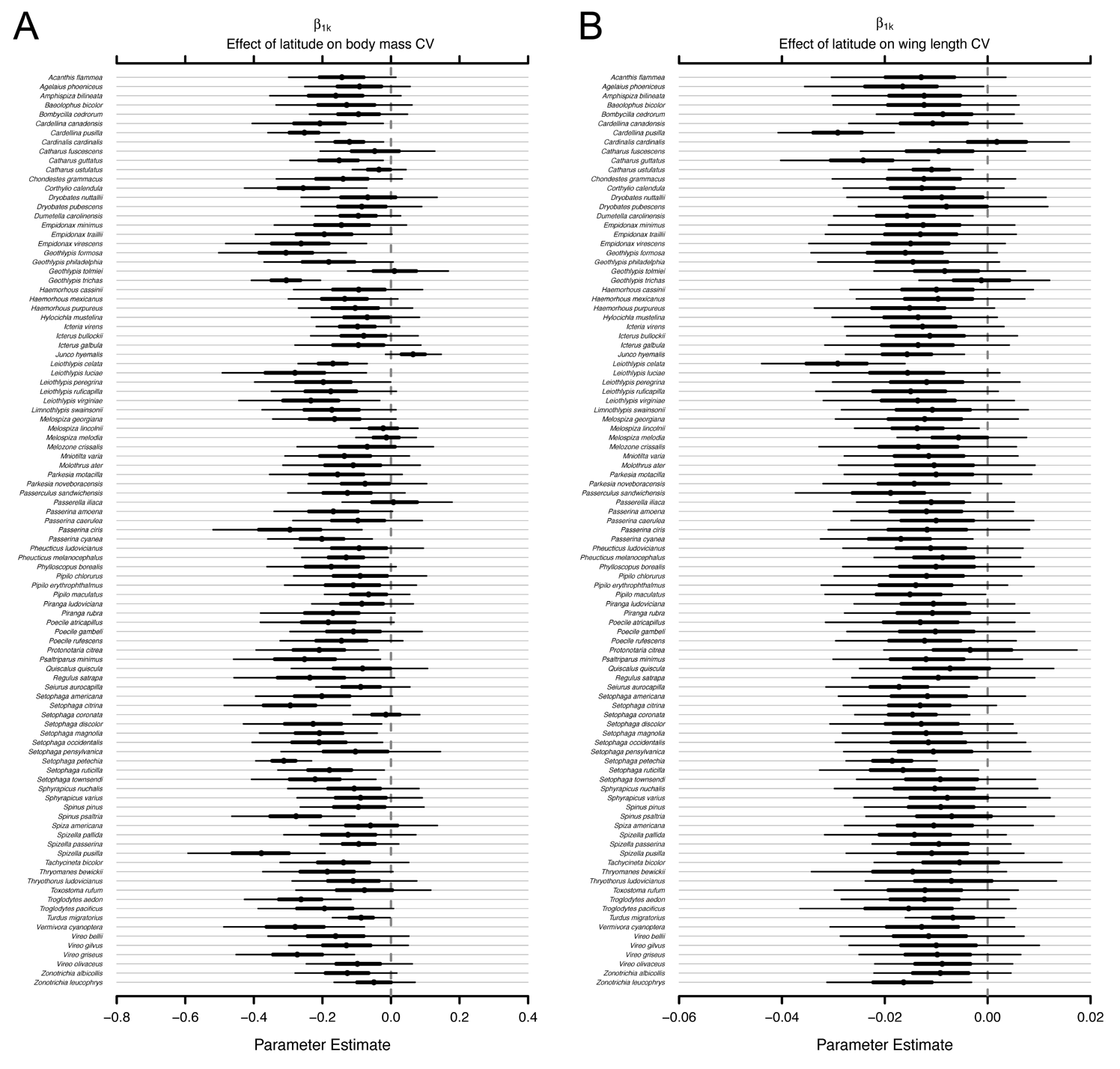


**Figure S1**


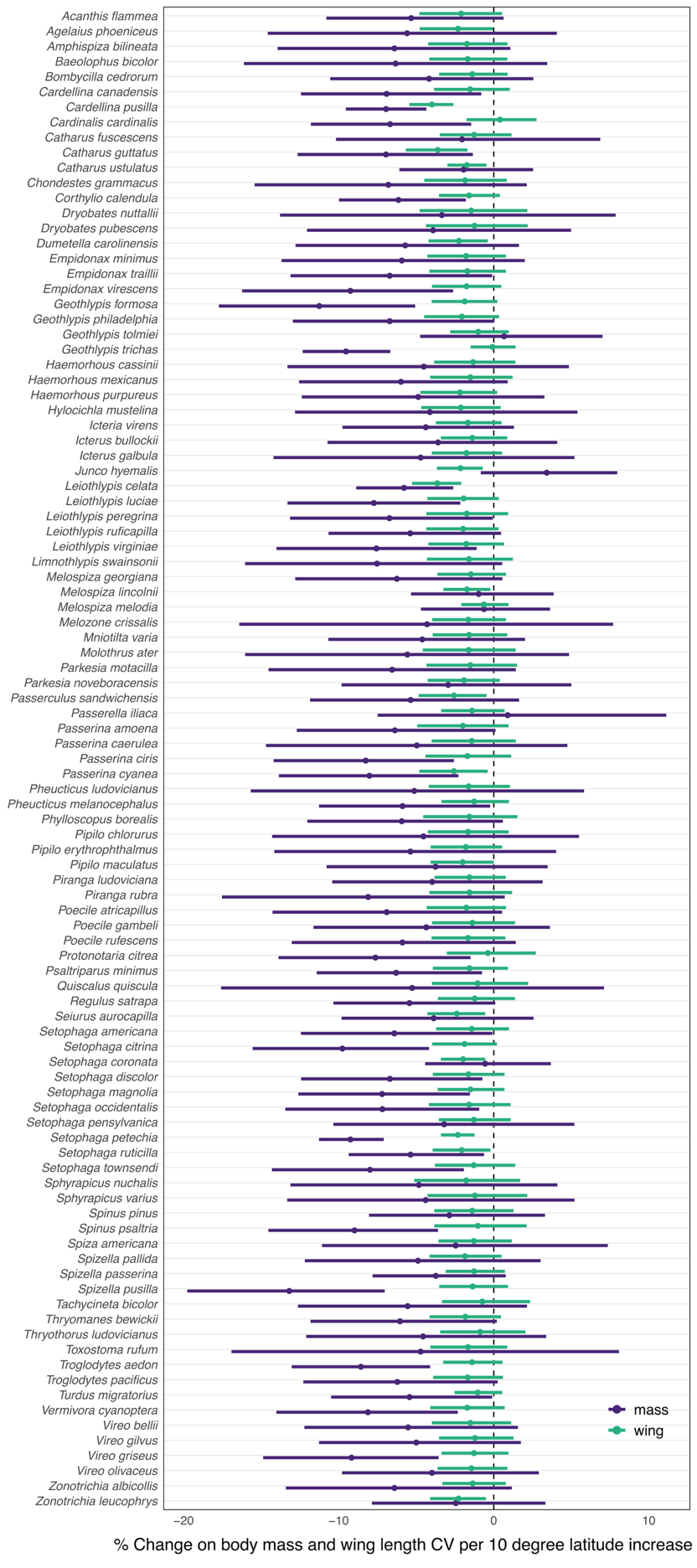


**Figure S2**


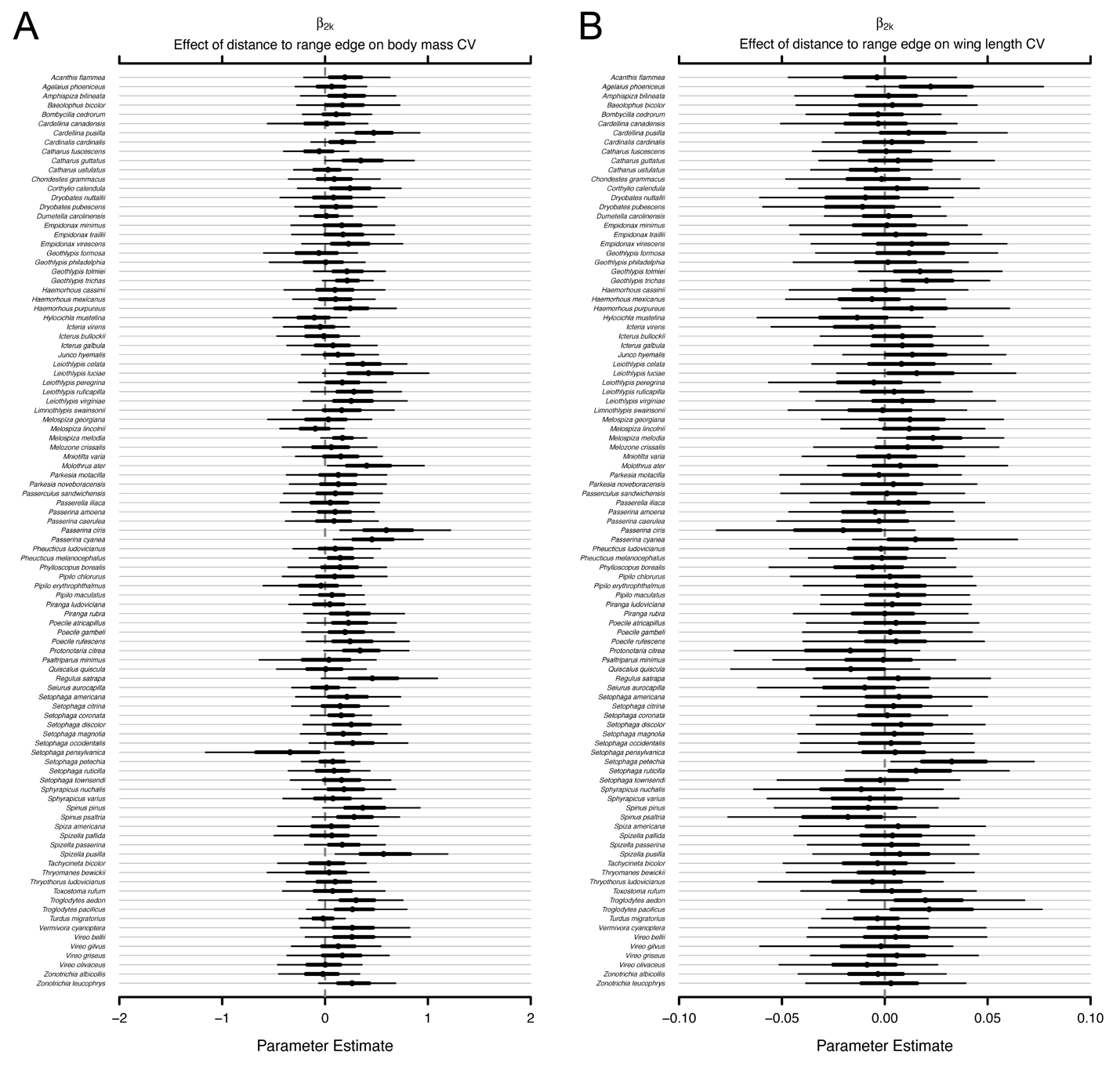


**Figure S3**

**
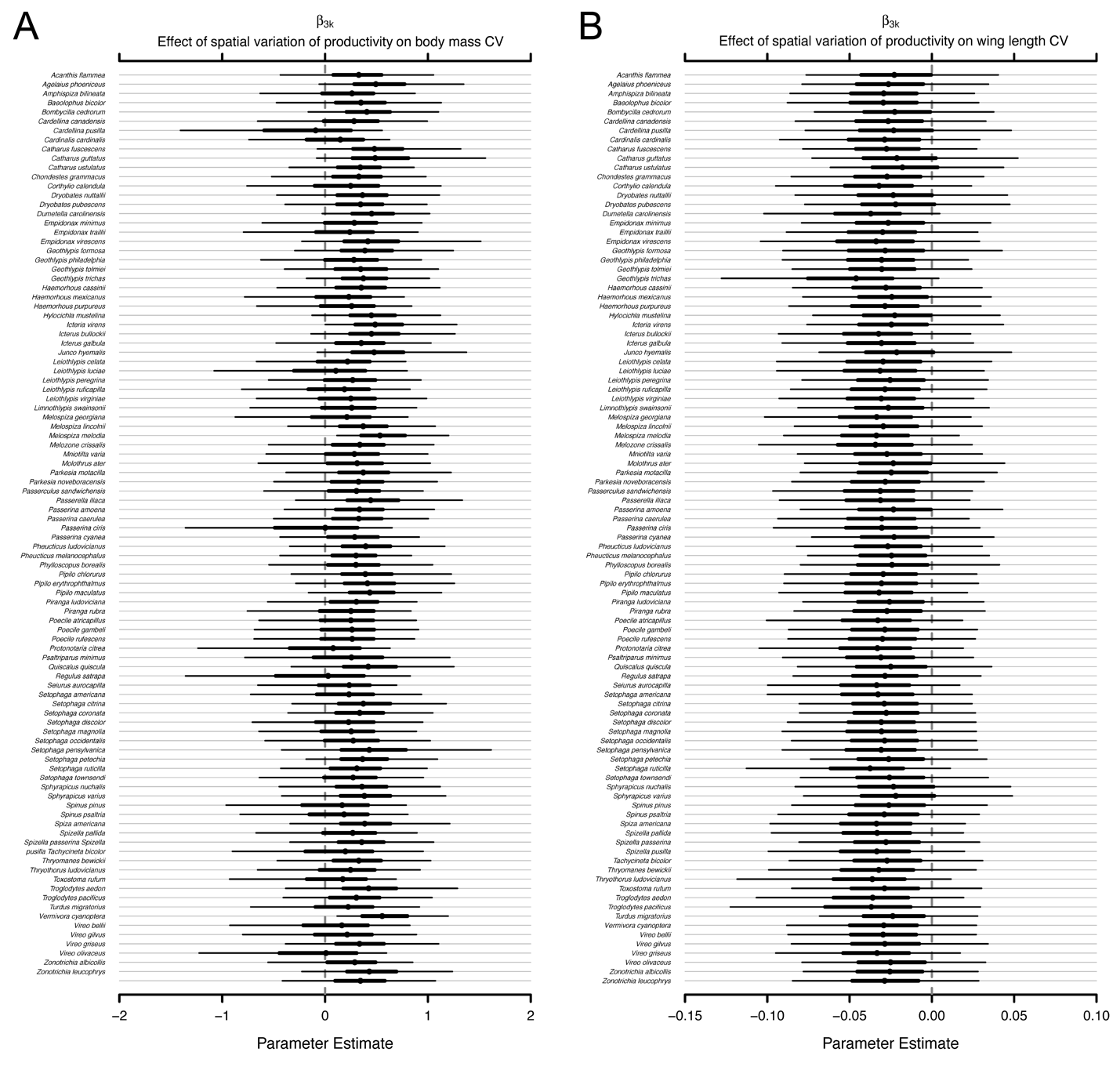
**

**Figure S4**

**
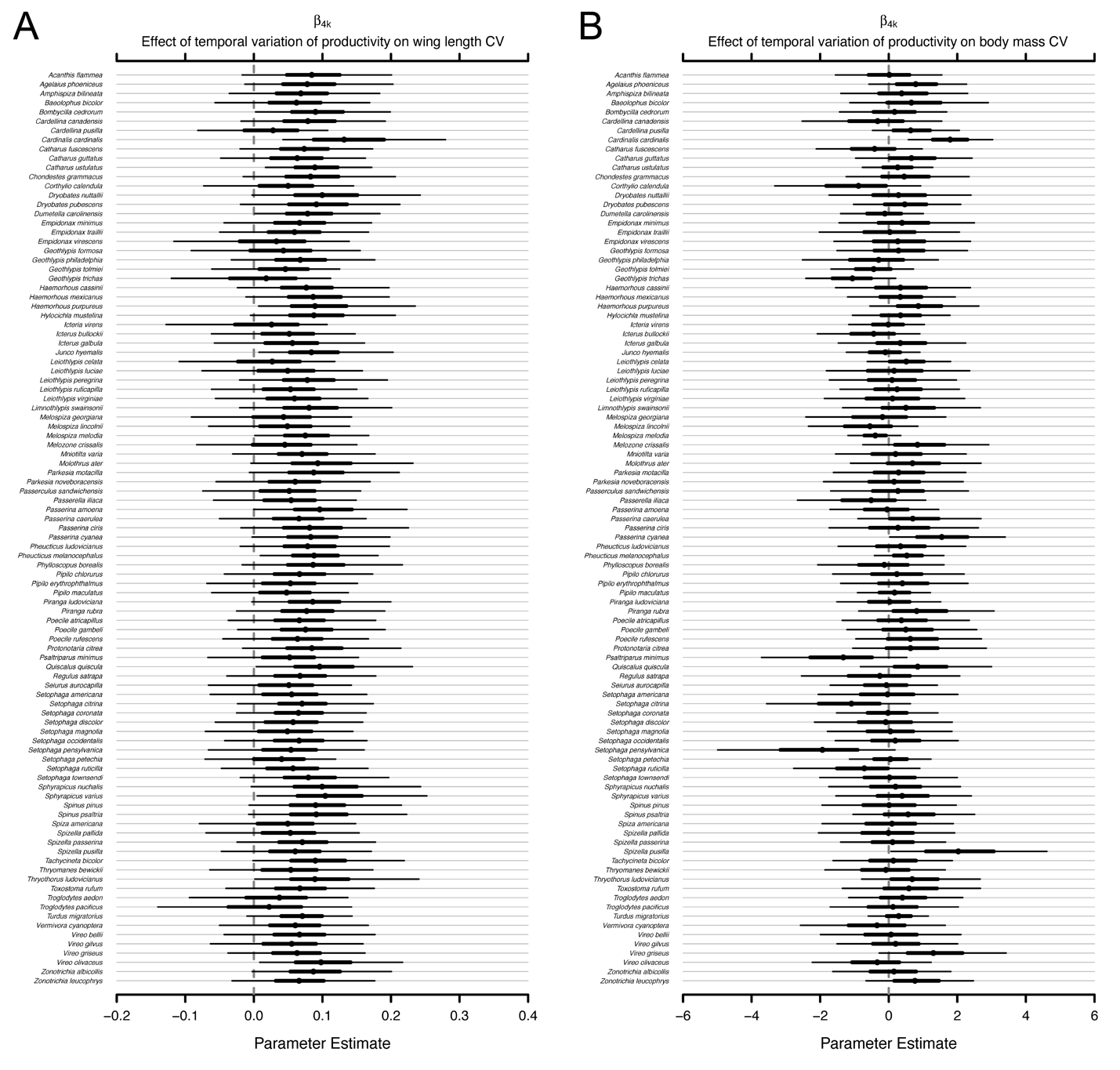
**

**Figure S5**

**
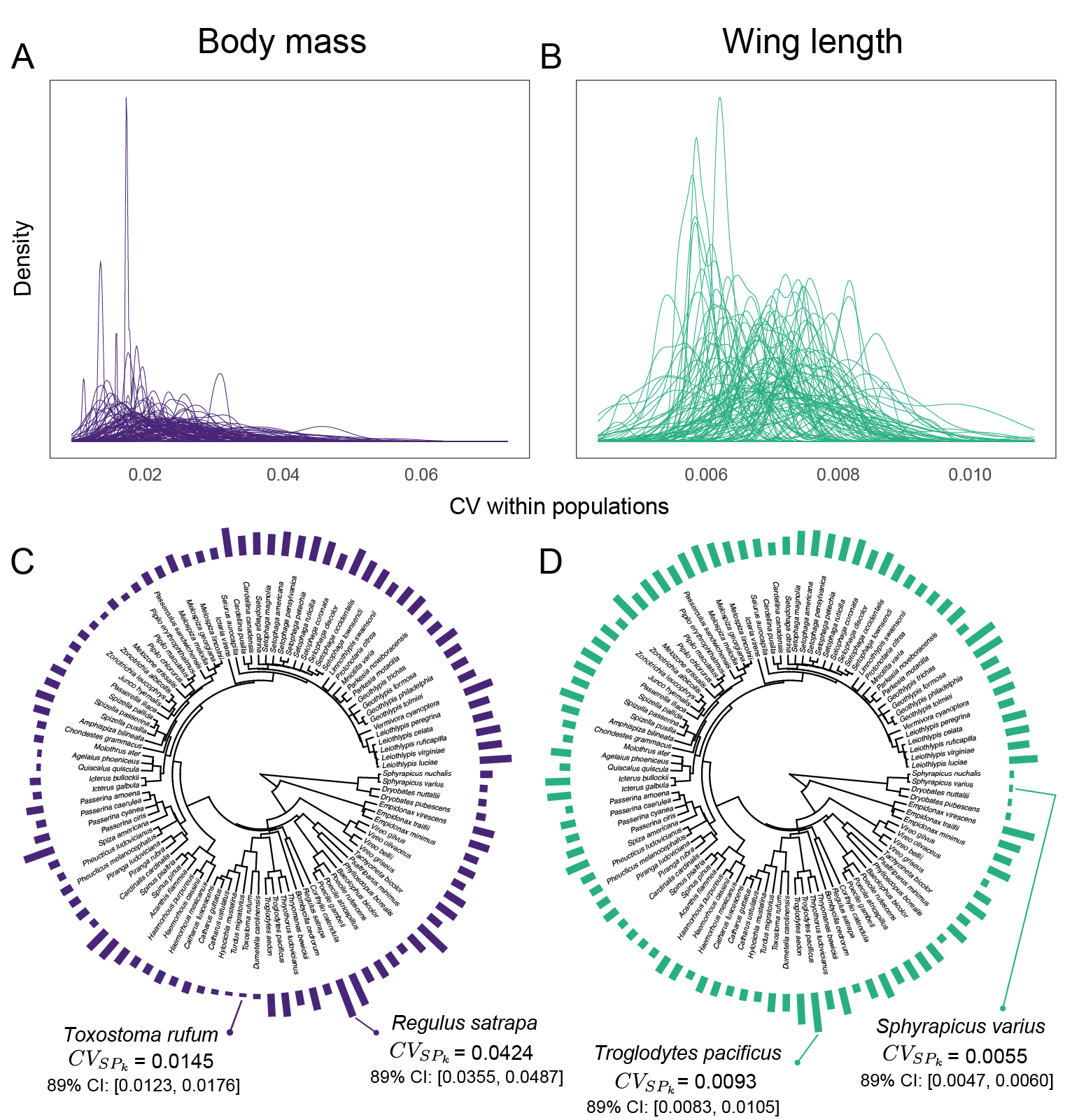
**

**Figure S6**

**
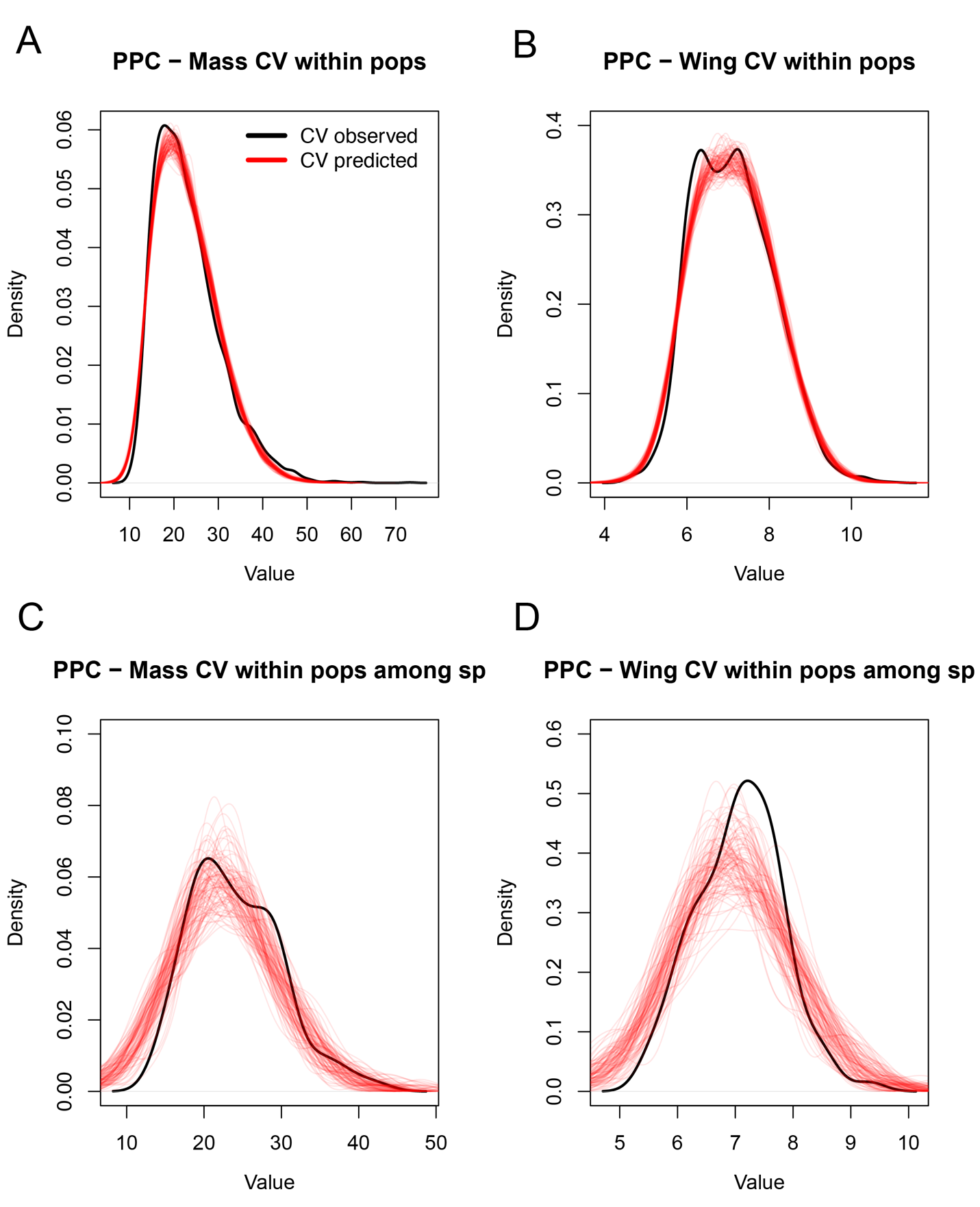
**

**Figure S7**
